# Screening and Identification of APOC1 as a Novel Potential Biomarker for Differentiate of *Mycoplasma pneumoniae* in Children

**DOI:** 10.3389/fmicb.2016.01961

**Published:** 2016-12-15

**Authors:** Jieqiong Li, Lin Sun, Fang Xu, Hui Qi, Chen Shen, Weiwei Jiao, Jing Xiao, Qinjing Li, Baoping Xu, Adong Shen

**Affiliations:** MOE Key Laboratory of Major Diseases in Children, National Key Discipline of Pediatrics (Capital Medical University), National Clinical Research Center for Respiratory Diseases, Beijing Key Laboratory of Pediatric Respiratory Infection Diseases, Beijing Pediatric Research Institute, Beijing Children's Hospital, Capital Medical UniversityBeijing, China

**Keywords:** plasma proteins, *Mycoplasma pneumoniae*, children, label-free quantitative proteomics, APOC1

## Abstract

**Background:** Although *Mycoplasma pneumoniae* (MP) is a common cause of community-acquired pneumonia (CAP) in children, the currently used diagnostic methods are not optimal. Proteomics is increasingly being used to study the biomarkers of infectious diseases.

**Methods:** Label-free quantitative proteomics and liquid chromatography-mass/mass spectrometry were used to analyze the fold change of protein expression in plasma of children with MP pneumonia (MPP), infectious disease control (IDC), and healthy control (HC) groups. Selected proteins that can distinguish MPP from HC and IDC were further validated by enzyme-linked immunosorbent assay (ELISA).

**Results:** After multivariate analyses, 27 potential plasma biomarkers were identified to be expressed differently among child MPP, HC, and IDC groups. Among these proteins, SERPINA3, APOC1, ANXA6, KNTC1, and CFLAR were selected for ELISA verification. SERPINA3, APOC1, and CFLAR levels were significantly different among the three groups and the ratios were consistent with the trends of proteomics results. A comparison of MPP patients and HC showed APOC1 had the largest area under the curve (AUC) of 0.853, with 77.6% sensitivity and 81.1% specificity. When APOC1 levels were compared between MPP and IDC patients, it also showed a relatively high AUC of 0.882, with 77.6% sensitivity and 85.3% specificity.

**Conclusion:** APOC1 is a potential biomarker for the rapid and noninvasive diagnosis of MPP in children. The present finding may offer new insights into the pathogenesis and biomarker selection of MPP in children.

## Introduction

*Mycoplasma pneumoniae* (MP), the smallest free-living organism, is a common cause of upper and lower respiratory tract infections (Sanchez-Vargas and Gomez-Duarte, 2008). MP pneumonia (MPP) causes up to 40% of community-acquired pneumonia (CAP) in children and this is even higher ratio during epidemics. Although it is a self-limiting disease, some cases develop into refractory or fulminant pneumonia that can threaten the lives of children (Waites and Talkington, 2004).

The pathophysiology of MP infection is complex and the underlying molecular mechanisms are reported to be associated with many proteins. MP infection is thought to influence the expression of associated proteins, which are released into the bloodstream through different pathways (Covani et al., 2008; Sun et al., 2008; Li et al., 2014). Plasma proteins including cytokines, growth factors, extracellular matrix proteins, and other soluble mediators are essential for MP infection. In terms of pediatric MPP diagnosis, culture and serological tests are insensitive, time-consuming, and cross-reactive in children (Daxboeck et al., 2003; Long et al., 2012); therefore, they are not appropriate for the rapid and accurate detection of MP infection in clinical practice.

In general, detecting biomarkers in the plasma is a useful auxiliary method to diagnosis disease (Chen et al., 2013; Meyer Sauteur et al., 2014; Shu et al., 2015). Recently, advances in high-throughput technologies, such as proteomics, have made the analysis of plasma proteins possible (Li et al., 2014). Proteomic analysis using a label-free protocol is increasingly being performed for biomarker selection. Based on the principle that a special mixture of plasma proteins present different characterizations, this technique has been widely used in many diseases including infectious diseases (Papadopoulos et al., 2004; Ren et al., 2004; Agranoff et al., 2006; Hodgetts et al., 2007), cancer (Engwegen et al., 2006), and vascular disease (Pinet et al., 2008; Zhang et al., 2008; Hong et al., 2009). Although many protein biomarkers of MPP have been indicated by proteomics, specific proteins that can be used to discriminate MPP from other infection diseases, especially in children, have not been fully elucidated.

In this study, we analyzed the fold change of protein expression of children with MPP, infectious disease controls (IDC), and healthy controls (HC) using label-free quantitative proteomics and liquid chromatography-mass/mass spectrometry (LC-MS/MS). Proteins identified that could distinguish MPP from HC and IDC were further validated by enzyme-linked immunosorbent assay (ELISA). The aim of this study was to screen potential protein biomarkers in plasma from children that could be used to distinguish MPP from HC and IDC.

## Materials and methods

### Patients and controls

This study was performed in the Beijing Children's Hospital from November 2014 to September 2015. During the first period, a total of 20 children hospitalized with a final diagnosis of MPP confirmed in serum samples using PCR and ELISA were enrolled. Symptoms of children included fever, acute respiratory symptoms (cough, tachypnoea, difficult breathing) or both (Tamura et al., 2008; Wang et al., 2014). Seventeen other children defined as IDC were collected accordingly and had symptoms including respiratory symptoms and negative MPP immunoglobulin (Ig) M (<1:80) to exclude MPP. HC group subjects (*n* = 20) were recruited from children undergoing physical examination in Beijing Children's Hospital from November 2014 to May 2015. Patients with immunosuppression and those who received immunosuppressive therapy were excluded. All the groups were matched by age, gender, and ethnicity.

### Protein extraction

Human plasma with the removal of IgG, IgA, albumin, antitrypsin, haptoglobin, and transferrin, were mixed together for each group and divided into three tubes which were tested respectively. Each mixed sample was suspended with phosphate buffered saline (PBS, 50 μL), centrifuged at 10,000 × g for 30 min in 4°C, and suspended in 100 μL lysis buffer (7 M urea, 2 M thiourea). After being centrifuged at 40,000 × g for 30 min, proteins were extracted by ultrasonic sonication and precipitated with trichloroacetic acid for 30 min on ice. Then, samples were diluted with 50 mM NH_4_HCO_3_ to a final concentration of 0.5 mg/mL, and each sample was mixed with DTT (5 μL, 1 mol/L) at 37°C for 60 min, diluted with IAA (20 μL, 1 mol/L) for 60 min in the dark, and digested with trypsin at 37°C for 12 h. The digested supernatant fractions were used for LC-MS/MS analysis (Lee et al., 2015).

### LC-MS/MS analysis

Peptide mixtures were subjected to nano-liquid chromatography associated with MS for protein identification. All of the mass analyses were performed using a LC-MS/MS system, which consisted of an Agilent 1100 quaternary HPLC (Agilent, EASY-nLC1000, USA) and a Q-Exactive mass spectrometer (Thermo Finnigan, Germany) with the application of a distal 180°C source temperature. An RP trap column (Thermo EASY column SC200, 150 μm × 100 mm) and a C18 reverse-phase column (Thermo EASY column SC001 traps, 150 μm × 20 mm) was used for desalting and separating samples, respectively. Mobile phase A consisted of HPLC-grade water containing 0.1% formic acid (FA), and phase B consisted of 84% HPLC-grade acetonitrile (ACN) containing 0.1% FA. The analytical separation was run at a flow rate of 400 nl/min by using a linear gradient of phase B as follows: 0–45% for 100 min, 45–100% for 8 min, and 100% for 12 min. Each LC-MS/MS analysis was repeated three times to reduce technical variation (Lee et al., 2015).

### Bioinformatics analysis

For protein identification, we used the Mascot 2.1 program (Matrix science) to search fragmentation spectra against a human database. For full MS or MS/MS spectra searches, an error of six parts per million (ppm) or 20 ppm was set, respectively, and two missed cleavages were allowed. Peptide mass tolerance was set to 0.5 Da, fragment mass tolerance was set to 10 ppm. Variable modification was oxidation of methionine, static modification was carbamidomethylation of cysteine. Protein identification was considered valid if at least one peptide and the *p* < 0.05, the proteins not satisfying these defined criteria were rejected, the threshold for accepting MS/MS spectra was false discovery rate (FDR) 0.05. The Maxquant MS Analysis Software was used to estimate the fold-changes in the level of identified proteins between the three groups.

A Web-accessible resource (Version 2.0) was used to determine the over-representation of Gene Ontology (GO) categories, which was classified on the basis of cellular component (CC), molecular function (MF), and biological process (BP) (Zhang et al., 2014; Wu et al., 2016). Signaling pathway analysis was performed using tools from the Kyoto Encyclopedia of Genes and Genome (KEGG) database (http://www.genome.jp/kegg/pathway.html) (Kanehisa et al., 2004). A network model of protein interactions according to known protein-protein interactions was made using Retrieval of Interacting Genes (STRING) 9.05.

### ELISA

ELISA was used to quantify the concentrations of the selected plasma proteins. Human alpha-1-antichymotrypsin precursor (SERPINA3) (RayBiotech), human kinetochore-associated protein 1 (KNTC1) (Dldevelop), human apolipoprotein C-I precursor (APOC1) (RayBiotech), human annexin A6 isoform 2 (ANXA6) (Abnova), and CASP8 and FADD-like apoptosis regulator isoform 6 (CFLAR) (Dldevelop) ELISA kits were used to measure the changes in plasma protein levels in the MPP (*n* = 85), HC (*n* = 95), and IDC (*n* = 75) groups. ELISAs were performed according to each kit's instructions.

### Statistical analysis

Each experiment was independently repeated at least three times. Data were expressed as the mean ± *SD* and evaluated by Student's *t*-test. *P* < 0.05 was considered statistically significant.

Statistical analysis was performed using MetaboAnalyst 3.0 (Biomarker Analysis).

Classical univariate receiver operating characteristic (ROC) curve analyses to generate ROC curves, calculate the area under the curve (AUC) and 95% confidence intervals, compute optimal cutoffs for any given feature, and to generate performance tables for sensitivity, specificity, and confidence intervals at different cutoffs were performed. ROC curve analyses were performed based on three multivariate algorithms—support vector machines, which were used to assess the diagnostic value of candidate proteins.

### Ethics statement

This research was approved by the Ethics Committee of Beijing Children's Hospital (Supplementary Table 1). Written informed consent was collected from the children or the guardians of children. In accordance with the Declaration of Helsinki and the local ethics committee requirements, the plasma collection protocol in this research was based on the following standards: (1) Potential risk of the minor discomfort was minimized by utilizing only well trained personnel for the procedures; (2) No charge; (3) Written informed consent was provided to the children or the guardians; (4) The participants had the right to decide whether or not to participate; (5) The participants in this study will not receive direct benefits from the proposed research. However, the benefits to society could be a potentially less expensive and faster way to diagnose a serious disease and begin treatment earlier than current diagnostic methods.

## Results

### Characteristics of the study population

During the selected period, of 20 children with MPP enrolled in our study, 11 (55%) were boys. The mean age of the patients in the MPP group was 6.9 years, ranging from 1.9 to 12.4 years. Among 17 children in the IDC group, the causative agents included *Streptococcus pneumoniae* (*n* = 2), Influenza B virus (*n* = 3), *Escherichia coli* (*n* = 3), *Mycobacterium tuberculosis* (*n* = 2), *Haemophilus* (*n* = 3), and *Epstein-Barr virus* (*n* = 4). Regarding patient demographics, no significant differences in age or gender were identified among the MPP, HC, and IDC groups.

During the verification period, 85 MPP children, 95 HC and 75 IDC were recruited for ELISA verification. No significant differences in age or gender were identified among these three groups (Table 1).

**Table 1:** **Demographic characteristic of the participants**.

	**Selected period**				**Verification period**			
	**MPP**	**HC**	**IDC**	***P*-value^a^**	**MPP**	**HC**	**IDC**	***P*-value^a^**
Sample size	20	20	17	–	85	95	75	–
Sex (%)								
Boy	11	11	9	0.302	42	48	39	0.315
Girl	9	9	8		43	47	36	
Age (years)^b^	6.9 ± 3.0	6.3 ± 3.5	5.6 ± 3.9	0.323	7.0 ± 3.0	7.0 ± 3.0	6.2 ± 5.6	0.082
Age range (years)	1.9–12.4	3–12	1–12	–	1.5–15.1	2.1–17.1	1.9–17.2	–

a *P-value among MPP, HC, and IDC*.

b *Data are presented as mean ± SD*.

### Label-free quantitative proteomic analysis

Label-free quantitative proteomics coupled with LC-MS/MS analysis were used to compare samples from the three groups (MPP, HC, and IDC). Based on the LC-MS/MS data, a total of 2777 proteins were identified.

**Figure 1:**
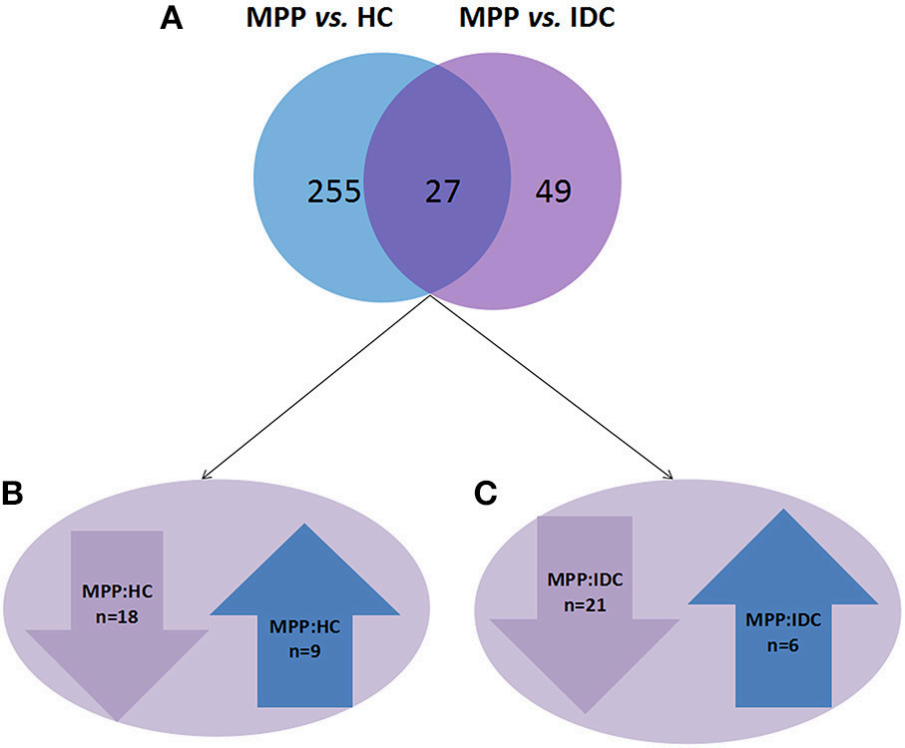
**Different proteins selected through LC-MS/MS analysis**. **(A)** 282 and 76 proteins differed in the MPP group (>2- or <0.5-fold), when compared to the HC group and IDC group, respectively. Of these, 27 proteins were overlapping. **(B)** 18 proteins were down-regulated (<0.5-fold) and 9 proteins were up-regulated (>2-fold) in MPP group when compared to HC group. **(C)** 21 proteins were down-regulated (<0.5-fold) and 6 proteins were up-regulated in MPP group when compared with IDC group.

The analysis results showed that 282 and 76 proteins differed between the MPP group (>2- or <0.5-fold) and the HC group and IDC group, respectively (Supplementary Tables 2 and 3). Of these, 27 overlapping proteins were further analyzed: 18 proteins were down-regulated (<0.5-fold) and 9 proteins were up-regulated (>2-fold) in the MPP group compared with the HC group. Twenty-one proteins were down-regulated (<0.5-fold) and six proteins were up-regulated (>2-fold) in the MPP group compared with the IDC group (Figure 1 and Table 2).

**Table 2:** **Proteins identified following LC-MS/MS of MPP different from healthy and disease control fractions**.

**Gi number**	**Protein name**	**Gene**	**Uniprot identifier**	**Mass**	**pI**	**Scores**	**MPP:HC**	**MPP:IDC**
gi|50659080	Alpha-1-antichymotrypsin precursor	*SERPINA3*	P01011	47.6	5.2	279.4	0.386	0.483
gi|7661960	Kinetochore-associated protein 1	*KNTC1*	P50748	250.6	5.6	127.2	0.069	0.264
gi|63998985	Mitogen-activated protein kinase kinase kinase 19 isoform 3	*MAP3K19*	Q56UN5	137.5	6.7	123.6	2.174	1.961
gi|93204879	PR domain zinc finger protein 15 isoform 1	*PRDM15*	P57071	169.2	9.6	123.4	0.233	0.439
gi|31791053	Zinc finger protein 804B	*ZNF804B*	A4D1E1	152.5	9.8	116.8	0.178	0.189
gi|4885583	rho-associated protein kinase 1	*ROCK1*	Q13464	158.1	5.6	113.3	2.941	0.431
gi|572882727	1-phosphatidylinositol 4,5-bisphosphate phosphodiesterase epsilon-1 isoform 3	*PLCE1*	Q9P212	256.9	6	106.8	0.188	2.000
gi|6912622	DNA repair and recombination protein RAD54B isoform 1	*RAD54B*	Q9Y620	102.9	9.4	98.7	0.300	8.333
gi|33620769	E3 ubiquitin-protein ligase RBBP6 isoform 1	*RBBP6*	Q7Z6E9	201.4	10.2	89.3	2.174	0.529
gi|108773808	Coiled-coil domain-containing protein 174	*CCDC174*	Q6PII3	53.9	6	84.6	0.361	0.266
gi|262118216	Coiled-coil domain-containing protein 88B precursor	*CCDC88B*	A6NC98	164.7	4.9	83.1	2.273	0.328
gi|140161498	Microtubule-associated tumor suppressor candidate 2 isoform a	*MTUS2*	Q5JR59	151.1	6.3	82.3	0.503	0.526
gi|44921615	Exocyst complex component 8	*EXOC8*	Q8IYI6	81.7	5.2	78.1	2.941	0.431
gi|356461016	Gem-associated protein 5 isoform 2	*GEMIN5*	Q8TEQ6	168.4	6.2	73.6	0.439	0.526
gi|4502157	Apolipoprotein C-I precursor	*APOC1*	P02654	9.3	9.3	73.5	2.083	2.273
gi|302129652	Annexin A6 isoform 2	*ANXA6*	P08133	72.4	5.3	71.3	0.154	0.103
gi|338753408	Transcription factor IIIB 90 kDa subunit isoform 5	*BRF1*	Q92994	61.8	5	60.8	0.455	0.372
gi|32967603	Bromodomain adjacent to zinc finger domain protein 1A isoform a	*BAZ1A*	Q9NRL2	178.6	6.2	59.6	0.538	0.227
gi|321267571	CASP8 and FADD-like apoptosis regulator isoform 6	*CFLAR*	O15519	41.3	7	51.9	0.538	0.493
gi|83700225	Potassium-transporting ATPase alpha chain 2 isoform 2	*ATP12A*	P54707	115.4	6.1	45.4	0.309	0.439
gi|390407643	cAMP-regulated phosphoprotein 21 isoform 4	*ARPP21*	Q9UBL0	88.5	6.5	41.1	0.439	0.422
gi|284925165	SUN domain-containing protein 1 isoform c	*SUN1*	O94901	76.4	6.2	40.4	2.326	2.778
gi|4507955	Transcriptional repressor protein YY1	*YY1*	P25490	44.7	5.8	38.1	2.128	0.124
gi|392050772	Zinc finger protein 850 isoform 2	*ZNF850*	A0A087X0M6	121.8	10.1	37.5	2.041	2.128
gi|149588534	Ataxin-7-like protein 3 isoform b	*ATXN7L3*	Q14CW9	38.6	6.8	36.6	0.278	0.407
gi|222352127	Protein sidekick-2 precursor	*SDK2*	Q58EX2	239.2	6.6	85.3	0.483	0.546
gi|378925630	Ubiquitin carboxyl-terminal hydrolase 17-like protein 10	*USP17L10*	C9JJH3	59.8	9.4	50.5	0.483	0.546

**Figure 2:**
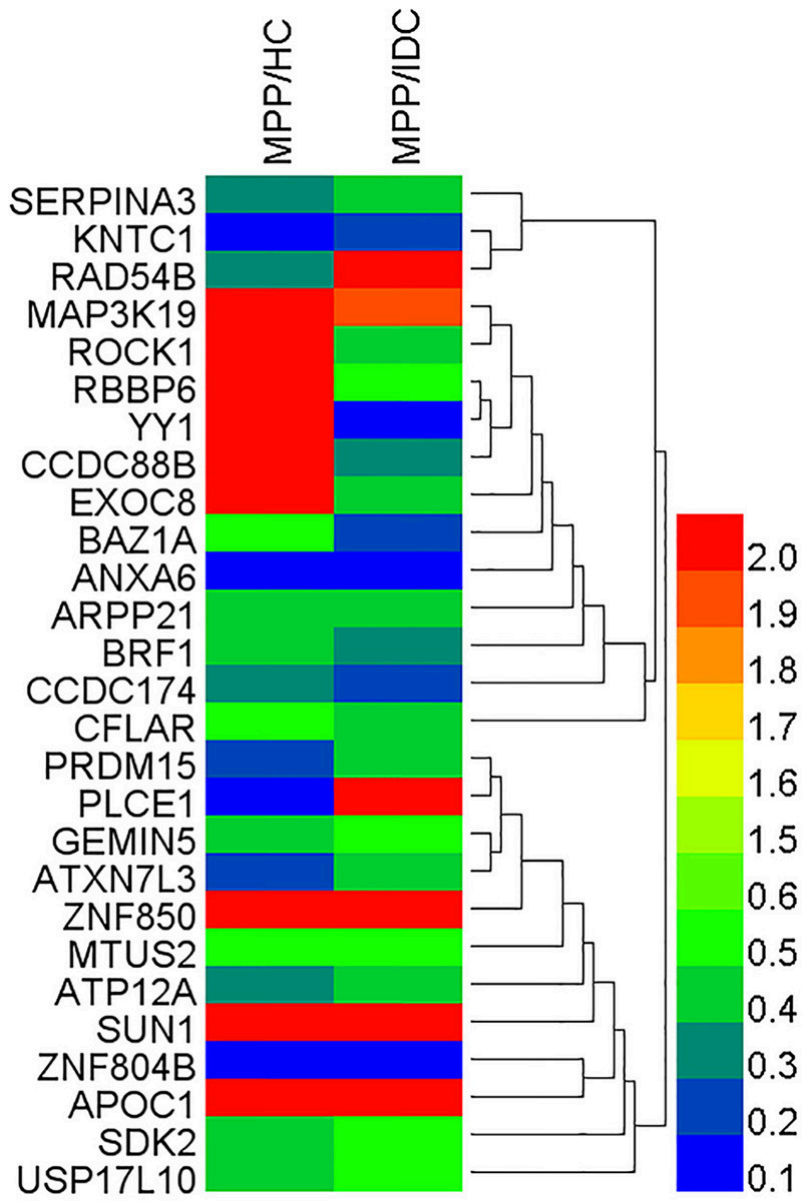
**Heat map of the 27 identified proteins**. The most striking area of up-regulation in TB patients is seen in the region where a series of protein peaks showed in red.

To reveal the bio-function relationship among the proteins, a hierarchical clustering based on Pearson correlation of variances was applied by R studio. Figure 2 shows the hierarchical clustering of 27 identified proteins, where an increasing red color shows increasing protein expression levels (Figure 2). The most striking area of up-regulation in MPP patients was indicated by a region with a series of protein peaks (red color).

### Bioinformatic analysis of differentially expressed proteins during MP infection

#### GO analysis

**Figure 3:**
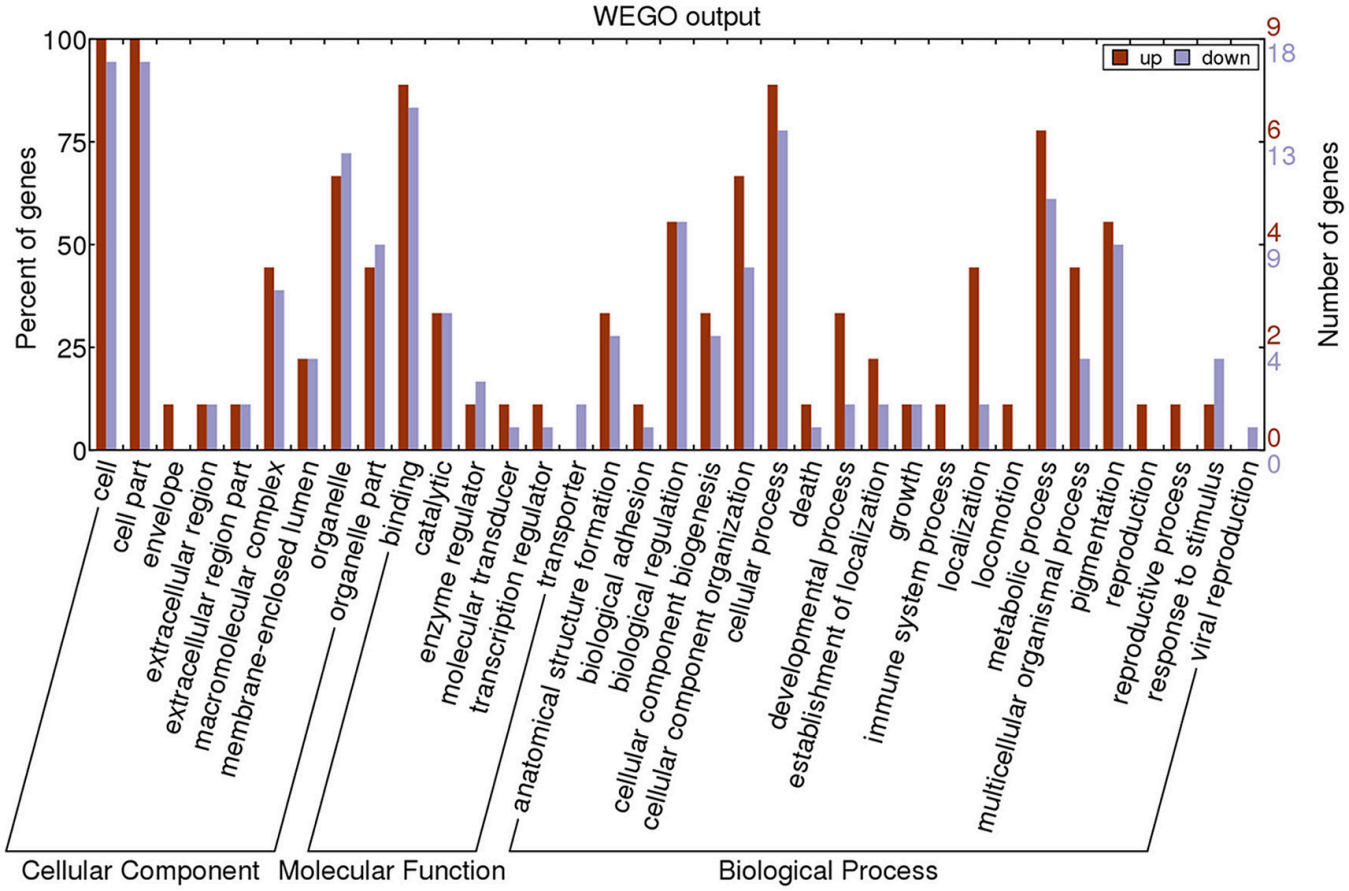
**Gene Ontology (GO) classification of differentially expressed proteins by label-free Quantitative Proteomics experiments between MPP and controls**. The differentially expressed proteins are grouped into three hierarchically structured GO terms: biological process, cellular component, and molecular function. The y-axis indicates the number and percent of proteins in each GO term.

GO annotation was used to analyze the functions of the 27 proteins, which were classified into three categories: CC, MF, and BP. To visualize the annotation of gene sets, WEGO was used to plot the distribution of GO annotations. The differentially identified proteins were subcategorized into 35 main hierarchically structured GO classifications including 9 CC, 6 MF, and 20 BP (Figure 3 and Supplementary Table 4).

The majority of proteins in the CC category had a cellular part and organelle distribution. The 27 proteins in the MF category were mainly related to binding, catalysis, enzyme regulator, molecular transducer, transcription regulator, and transporter. The 27 proteins in the BP category were mainly associated with cellular processes, metabolic processes, cellular component organization, biological regulation, and pigmentation. This analysis indicated that the identified proteins involved in these GO categories might have the most important roles in the MP infection process.

#### KEGG enrichment analysis

**Figure 4:**
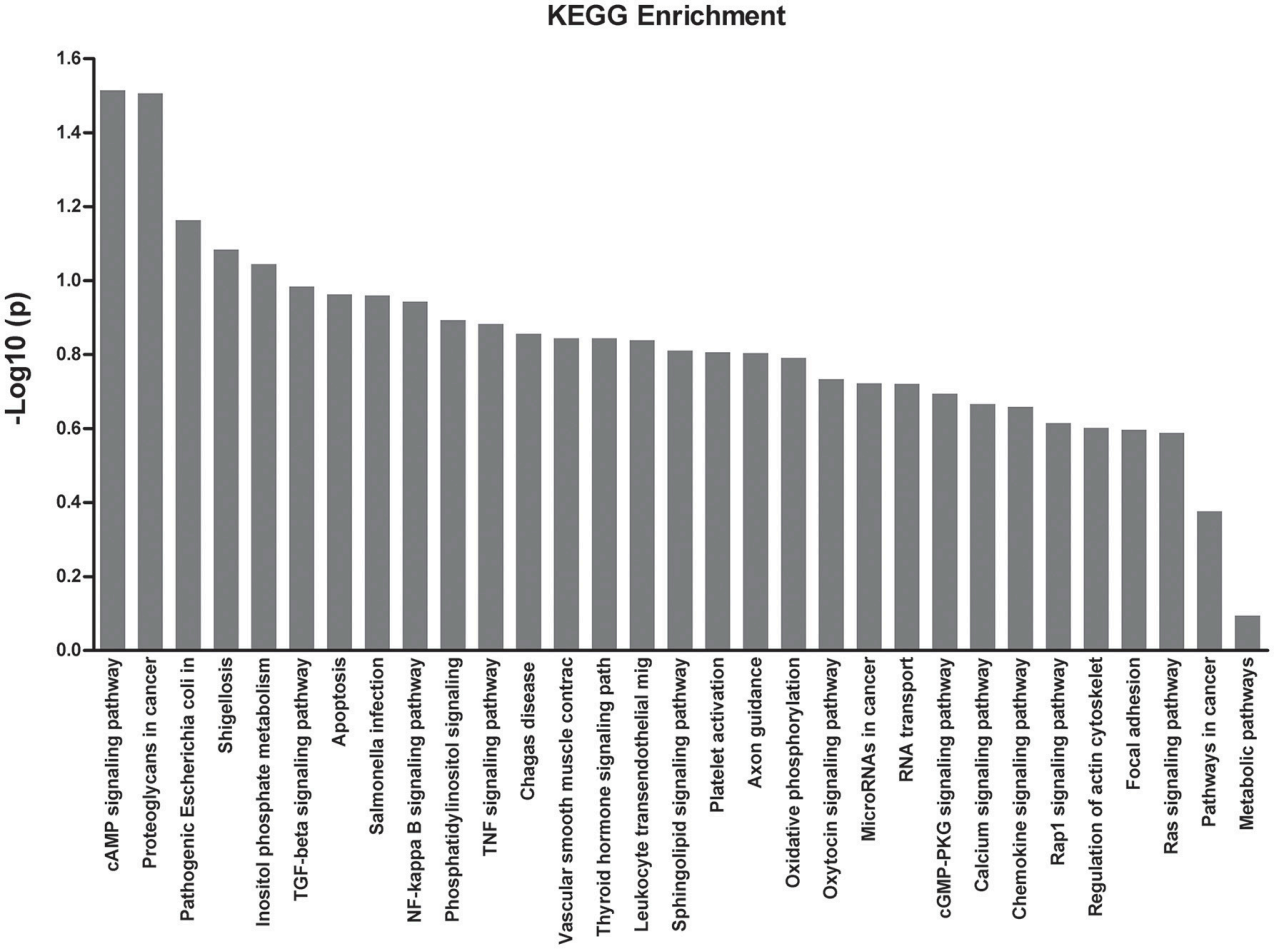
**KEGG enrichment analysis of the differential expressed proteins**.

To obtain more information with regards to the markers, a pathway analysis was conducted using KEGG. Using a standard of *P* < 0.05 and impact factor threshold >0, the pathway analysis results demonstrated that the cAMP signaling pathway (*P* = 0.026) and proteoglycans in cancer (*P* = 0.028) were significantly associated with MPP infection (Figure 4 and Supplementary Table 5).

### Identification of novel biomarkers for the differentiate of MPP

#### Validation of identified proteins by ELISA

**Figure 5:**
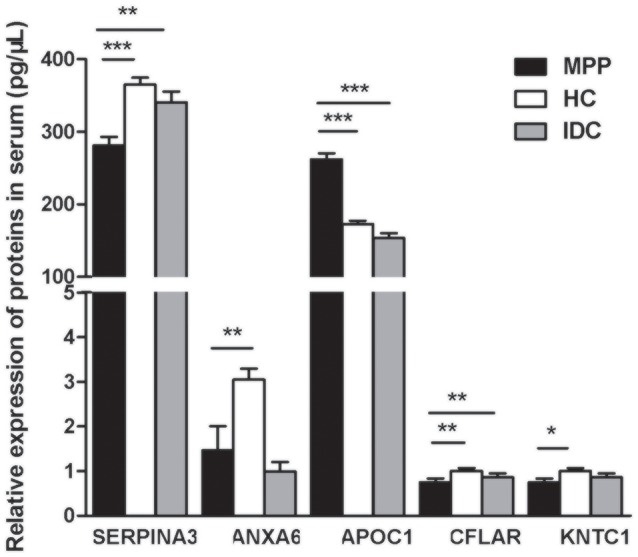
**Five selected proteins different between ATB and LTBI were analyzed by ELISA (MPP = 85, HC = 95, IDC = 75)**. Significant differences in SERPINA3, APOC1, and CFLAR levels were noted among all three groups and their ratios were consistent with the trends of proteomics results. Data are presented as means ± *SD*. ^*^p < 0.05; ^**^p < 0.01; ^***^p < 0.001

Based on the bioinformatics analysis, five potential candidate biomarkers (APOC1, SERPINA3, ANXA6, KNTC1, and CFLAR) were selected for verification (Figure 5 and Supplementary Table 6). The selection principles were based on the following standards: (1) high fold change; (2) representative in the 27 proteins; (3) associated with immunity or infectious diseases; and (4) present the same trends when compared with the HC and IDC.

Significant differences in SERPINA3, APOC1, and CFLAR levels were observed among all three groups and their ratios were consistent with the trends of the proteomic results (*P* < 0.001, 0.001, and 0.008, respectively). As shown in Figure 5, the level of APOC1 protein in MPP was markedly increased when compared with that in HC or IDC. In contrast, significantly lower levels of the two proteins (SERPINA3 and CFLAR) were observed in the MPP group compared with that in HC or IDC. Although the levels of ANXA6 and KNTC1 were lower in the MPP group compared with the HC group, there were no differences between the MPP and IDC patients.

#### Performance of each selected protein to differentiate of MPP

The AUC, sensitivity, and specificity of ROC curves calculated at optimal cutoffs for the three different proteins are shown in Table 3. A comparison of MPP patients and HC showed APOC1 had the largest AUC of 0.853, with 77.6% sensitivity and 81.1% specificity. The comparison of MPP and IDC patients showed APOC1 had a relatively high AUC of 0.882, with 77.6% sensitivity and 85.3% specificity. SERPINA3 and CFLAR had AUC-values of 0.739 and 0.524 when MPP patients were compared with HC, respectively, and they had lower AUC-values of 0.643 and 0.647 when compared between MPP and IDC patients, respectively. In addition, we combined the HC and IDC for further analysis. APOC1, SERPINA3, and CFLAR had AUC-values of 0.866, 0.698, and 0.578 when MPP was compared with HC plus IDC, respectively. Our results showed that the APOC1 in MPP group presented the best AUC among these three proteins when compare with HC, IDC, or HC combined with IDC.

**Table 3:** **AUC, sensitivity, and specificity for ROC curves calculated at optimal cutoff for SERPINA3, APOC1, and CFLAR**.

**Significant proteins by comparison groups**	**AUC**	**95% CI**	**Sensitivity (%)**	**Specificity (%)**	***P*-value**	**Log_2_ FC**
**MPP vs. HC**						
APOC1	0.853	0.796–0.907	77.6	81.1	8.81E–18	0.56
SERPINA3	0.739	0.662–0.818	77.6	68.4	1.38E–7	−0.34
CFLAR	0.524	0.436–0.61	77.6	42.1	7.56 E–3	0.12
**MPP vs. IDC**						
APOC1	0.882	0.834–0.93	77.6	85.3	1.37E–18	0.78
SERPINA3	0.643	0.558–0.735	72.9	65.3	2.15E–3	−0.33
CFLAR	0.647	0.55–0.733	81.2	50.7	1.54 E–3	−0.66
**MPP vs. (HC** + **IDC)**						
APOC1	0.866	0.816–0.915	77.6	82.9	1.34E–26	0.55
SERPINA3	0.698	0.624–0.763	77.6	64.7	1.42E–6	−0.05
CFLAR	0.578	0.514–0.648	71.8	54.1	3.74 E–3	−0.64

#### Sensitivities and specificities of combinations of selected proteins

Using the same optimal cutoffs for each protein, we calculated the sensitivities and specificities of combinations of two or three selected proteins to differentiate MPP. As shown in Table 4, although the specificities of combined proteins were generally higher than APOC1, the sensitivities of the combined proteins were lower than this best protein for discriminating MPP patients from HC or IDC patients. Combined with the performance of each selected protein to differentiate of MPP, the results showed that APOC1 alone was the best choice in children.

**Table 4:** **Sensitivities and specificities of combinations of two or three proteins for differentiation between MPP and HC, IDC, or HC + IDC**.

**Proteins**	**MPP vs. HC**		**MPP vs. IDC**		**MPP vs. HC + IDC**	
	**Sensitivity (%)**	**Specificity (%)**	**Sensitivity (%)**	**Specificity (%)**	**Sensitivity (%)**	**Specificity (%)**
APOC1 + SERPINA3	76.5	87.4	76.5	85.3	77.7	82.9
APOC1 + CFLAR	75.3	82.1	76.5	81.3	75.3	81.8
SERPINA3 + CFLAR	75.3	68.4	74.1	58.7	75.3	67.1
SERPINA3 + APOC1 + CFLAR	74.1	84.2	78.8	81.3	77.7	83.5

## Discussion

Immune responses, induced by MP infection, have an important effect on pathogenic mechanisms and protein expression. Traditionally, proteins appear in the circulation through many different mechanisms including secretion after subjects are infected by a pathogen, when production is stimulated by antigens, or by direct secretion by MP (Li et al., 2014). Recently, high-throughput technologies have been used to investigate protein expression, and therefore, the accurate detection and quantification of these MP-associated proteins can be used as potential diagnostic markers.

Previous methods used to detect MPs had limitations (Daxboeck et al., 2003; Long et al., 2012; Yuta et al., 2014; Katsushima et al., 2015). In clinical practice, co-infections are common in children with pneumonia. Therefore, in this study, HC and IDC were both used as controls. By using label-free quantitative analysis, we analyzed plasma proteins among MPP, HC, and IDC individuals. This proteomic approach allowed the measurement of changes in protein expression, which might be used to “forecast” disease pathogenesis. As reported here, 282 and 76 proteins were identified in the MPP group compared with the HC and IDC groups, of which 27 were overlapping. In addition, WEGO was used to analysis the function of the 27 different proteins. Here, we observed that a majority of the proteins had a cellular distribution and organelles. They were mainly related to binding, catalysis, enzyme regulator, molecular transducer, transcription regulator, and transporter. They mostly participated in cellular processes, metabolic processes, cellular component organization, biological regulation, and pigmentation, all of which are thought to be essential in the pathogenesis of MP.

Among the 27 proteins identified, five potential candidate biomarkers (SERPINA3, KNTC1, APOC1, ANXA6, and CFLAR) were selected for verification because they correlated with immune responses in other infectious diseases. However, ELISA showed that only the levels of SERPINA3, APOC1, and CFLAR were significantly different among all three groups and their ratios were consistent with the trends of the proteomics analyses. Using the same optimal cutoffs for each protein, we analyzed the sensitivities and specificities of different combinations of two or three of the different proteins for the diagnosis of MPP. The specificities of combined proteins were generally higher than APOC1, while the sensitivities of combined proteins were generally lower than the best protein for differentiating MPP patients and HC or IDC patients. In summary, our results showed that APOC1 alone was the best choice for differentiate of MPP in children. APOC1 had the best sensitivity and specificity. To the best of our knowledge, the sensitivity and specificity of the MP-specific antigens currently used are poor when used as a diagnostic test based on ineffective antibody detection (Zhang et al., 2014). Li et al. (2014) used label-free quantitative proteomics to demonstrate that IL-33 production in A549 cells was increased when they were infected with MP. They also found that IL-33 levels were higher in MPP patients with sensitivity and specificity accuracy of 70.0 and 73.3%, respectively (Li et al., 2014). However, larger sample sizes are needed for further identification and quantification.

MP is a unique cell wall deficient and cholesterol requiring bacteria (Berbée et al., 2005; Ko et al., 2014). Cholesterol metabolism is essential for MP growth and infection. APOC1 is a 6.6-kDa plasma protein that inhibits receptor-mediated lipoprotein clearance (Shulman et al., 1975). It is secreted by many organs, including the liver, spleen, and lung, and is secreted into the blood (Lauer et al., 1988). Therefore, the plasma level of APOC1 is associated with cholesterol metabolism and MP infection status in humans. Lipid metabolism is one of the essential changes in many infectious diseases, including MPP (Katsel et al., 2007; Xiang et al., 2007). Furthermore, many cholesterol metabolites have been analyzed by studies with the aim of screening and identifying early biomarkers for infectious disease (Leoni et al., 2006; Bach et al., 2009; Gilch et al., 2009). Several genes associated with cholesterol metabolism and lipid biosynthesis were reported to be up-regulated in serum or plasma (Brown et al., 2005). Because APOC1 activates cholesterol metabolism (Barbisin et al., 2014), its up-regulation leads to an increase in cholesterol biosynthesis, consistent with the concomitant presence of MP infectious disease.

Our study demonstrated that APOC1 alone is a potential biomarker for differentiate of MPP in children. This was an exploratory study to detect novel plasma proteomics biomarkers and further studies are required. MPP causes up to 40% of CAP in children and biomarker selection requires a large sample verification. Clearly, this should be the focus of further investigations.

In conclusion, 27 potential MPP plasma biomarkers were identified using LC-MS/MS. After ELISA verification, APOC1 was identified with satisfactory sensitivity and specificity to discriminate MPP from HC or IDC, and represents a viable approach for the differentiate of MPP.

## Author contributions

JL and AS designed the experiments. JL and LS performed the experiments and analyzed the data. FX, WJ, and HQ provided technical support for the experiments. AS, JX, and CS provided comments and technical advice. JL, LS, and AS wrote the manuscript. JL, BX, and QL revised the manuscript. All authors discussed the results, commented on the manuscript, and agreed to be accountable for all aspects of the work in ensuring that questions related to the accuracy or integrity of any part of the work are appropriately investigated and resolved.

## Funding

This work was supported by grants from Beijing Natural Science Foundation (No. 7164257), Capital Health Research and Development of Special Grant (No. 2016-1-2092), the BeiJing Talents Fund (No. 2014000021469G244), and collaborative Innovation Center of Infectious Diseases (No. PXM2014_014226_000011).

### Conflict of interest statement

The authors declare that the research was conducted in the absence of any commercial or financial relationships that could be construed as a potential conflict of interest.
