# Supplementary material for: Screening and Identification of APOC1 as a Novel Potential Biomarker for Differentiate of Mycoplasma pneumoniae in Children
Source: Front Microbiol. 2016 Dec 15;7:1961. doi: 10.3389/fmicb.2016.01961 (PMC5156883; doi:10.3389/fmicb.2016.01961)
Supplement: Supplementary file 1 [file Table1.DOCX]

**Supplementary 1: The Ethics committee approval**

Medical Ethics Committee of Beijing Children’s Hospital Affiliated to Capital Medical University

Ethics Approval of Clinical Research

Declare: The organization and procedures of the committee comply with GCP

principle and national legislation

Number: 2014-99

Tittle of the project: Multicenter study for diagnosis and treatment of community acquired pneumonia

Researcher: Kunlin Shen, A-dong Shen and her colleagues, Beijing Pediatric Research Institute

Place: Beijing Children’s Hospital Affiliated to Capital Medical University

Date: 28th October, 2014

Evaluation:

Qualifications of the applicant: pass

Research plan: pass

Informed consent: pass

Benefit and risk of the experimenter: pass

Medical record: N/A

Original medical record: pass

Severe adverse events: N/A

Signature of committee member attending the meeting: here are the signatures

Voting on the applicant: all committee members approve

Conclusion: approve

Signature of the committee chairman: here is the signature

Date: Date: 28th October, 2014

Stamp the committee’s seal: here is the seal
