# Supplementary material for: Screening and Identification of APOC1 as a Novel Potential Biomarker for Differentiate of Mycoplasma pneumoniae in Children
Source: Front Microbiol. 2016 Dec 15;7:1961. doi: 10.3389/fmicb.2016.01961 (PMC5156883; doi:10.3389/fmicb.2016.01961)
Supplement: Supplementary file 2 [file Table2.DOCX]

**Supplementary 2: Proteins identified following LC-MS/MS of MPP different from healthy control fraction.**

| Gi number | Protein name | Gene | Uniprot Identifier | Mass | pI | Scores | MPP:HC |
| --- | --- | --- | --- | --- | --- | --- | --- |
| gi\|40316910 | serum amyloid A-1 protein preproprotein | SAA1 | P0DJI8 | 13.5 | 6.4 | 225.4 | 0.052 |
| gi\|7661960 | kinetochore-associated protein 1 | KNTC1 | P50748 | 250.6 | 5.6 | 127.2 | 0.069 |
| gi\|37595756 | probable D-lactate dehydrogenase, mitochondrial isoform 2 precursor | LDHD | Q86WU2 | 52.1 | 6 | 46.8 | 0.078 |
| gi\|93102379 | low-density lipoprotein receptor-related protein 1B precursor | LRP1B | Q9NZR2 | 515.2 | 5 | 173.0 | 0.106 |
| gi\|134152694 | retinal guanylyl cyclase 2 | GUCY2F | P51841 | 124.8 | 7 | 61.4 | 0.140 |
| gi\|193804860 | synaptotagmin-like protein 4 | SYTL4 | Q96C24 | 76 | 9.8 | 33.4 | 0.140 |
| gi\|302129652 | annexin A6 isoform 2 | ANXA6 | P08133 | 72.4 | 5.3 | 71.3 | 0.154 |
| gi\|306482667 | myeloid leukemia factor 1 isoform 4 | MLF1 | P58340 | 23.1 | 10.3 | 41.9 | 0.154 |
| gi\|39995082 | tRNA (cytosine(34)-C(5))-methyltransferase isoform 1 | NSUN2 | Q08J23 | 86.4 | 6.3 | 62.1 | 0.168 |
| gi\|193788632 | dnaJ homolog subfamily C member 2 isoform 2 | DNAJC2 | Q99543 | 65.9 | 9.7 | 47.6 | 0.172 |
| gi\|10092615 | ethanolamine kinase 1 isoform A | ETNK1 | Q9HBU6 | 50.9 | 6.1 | 52.4 | 0.175 |
| gi\|187960098 | medium-chain specific acyl-CoA dehydrogenase, mitochondrial isoform b precursor | ACADM | P11310 | 47 | 9.4 | 57.7 | 0.175 |
| gi\|31791053 | zinc finger protein 804B | ZNF804B | A4D1E1 | 152.5 | 9.8 | 116.8 | 0.178 |
| gi\|33286446 | opioid growth factor receptor | OGFR | Q9NZT2 | 73.3 | 4.6 | 33.2 | 0.182 |
| gi\|301129207 | uridine-cytidine kinase-like 1 isoform 2 | UCKL1 | Q9NWZ5 | 59.4 | 8.9 | 72.4 | 0.187 |
| gi\|45580723 | haptoglobin-related protein precursor | HPR | P00739 | 39 | 6.7 | 886.3 | 0.188 |
| gi\|572882727 | 1-phosphatidylinositol 4,5-bisphosphate phosphodiesterase epsilon-1 isoform 3 | PLCE1 | Q9P212 | 256.9 | 6 | 106.8 | 0.188 |
| gi\|42716295 | UAP56-interacting factor isoform 1 | FYTTD1 | Q96QD9 | 35.8 | 12.3 | 92.2 | 0.188 |
| gi\|223468600 | leishmanolysin-like peptidase isoform 1 | LMLN | Q96KR4 | 78.1 | 7.3 | 58.7 | 0.200 |
| gi\|7705740 | DNA-directed RNA polymerases I and III subunit RPAC2 isoform 1 | POLR1D | Q9Y2S0 | 15.2 | 5.5 | 40.3 | 0.200 |
| gi\|375493536 | maternal embryonic leucine zipper kinase isoform 3 | MELK | Q14680 | 69.1 | 9.8 | 80.5 | 0.204 |
| gi\|578803140 | PREDICTED: serine/threonine-protein kinase VRK2 isoform X4 | VRK2 | Q86Y07 | 45 | 9.7 | 58.8 | 0.207 |
| gi\|334085299 | transmembrane protein 185B | TMEM185B | Q9H7F4 | 40.6 | 8.9 | 43.4 | 0.207 |
| gi\|30089916 | phosphofurin acidic cluster sorting protein 1 | PACS1 | Q6VY07 | 104.8 | 8.4 | 63.4 | 0.210 |
| gi\|110347443 | TATA element modulatory factor | TMF1 | P82094 | 122.8 | 4.7 | 86.4 | 0.216 |
| gi\|4506787 | ras GTPase-activating-like protein IQGAP1 | IQGAP1 | P46940 | 189.1 | 6.1 | 63.2 | 0.216 |
| gi\|390608639 | baculoviral IAP repeat-containing protein 2 isoform 2 | BIRC2 | Q13490 | 64.1 | 6 | 34.4 | 0.216 |
| gi\|63003897 | NF-kappa-B-repressing factor isoform 2 | NKRF | O15226 | 77.6 | 9.6 | 106.3 | 0.217 |
| gi\|38176300 | nestin | NES | P48681 | 177.3 | 4.2 | 44.6 | 0.217 |
| gi\|47106052 | coiled-coil domain-containing protein 103 isoform 1 | CCDC103 | Q8IW40 | 27.1 | 5.7 | 54.5 | 0.223 |
| gi\|8922886 | ATP-dependent RNA helicase DDX19A | DDX19A | Q9NUU7 | 53.9 | 6.2 | 61.6 | 0.225 |
| gi\|93204879 | PR domain zinc finger protein 15 isoform 1 | PRDM15 | P57071 | 169.2 | 9.6 | 123.4 | 0.233 |
| gi\|40549433 | putative tyrosine-protein phosphatase TPTE isoform beta | TPTE | P56180 | 62.4 | 9.2 | 60.2 | 0.234 |
| gi\|291167777 | transmembrane protease serine 4 isoform 5 | TMPRSS4 | Q9NRS4 | 43.7 | 4.8 | 46.2 | 0.239 |
| gi\|14249672 | protein phosphatase 1 regulatory subunit 16A | PPP1R16A | Q96I34 | 57.8 | 5.6 | 41.7 | 0.239 |
| gi\|93352558 | inactive rhomboid protein 2 isoform 2 | RHBDF2 | Q6PJF5 | 93.3 | 9.9 | 54.4 | 0.248 |
| gi\|7656861 | disintegrin and metalloproteinase domain-containing protein 18 isoform 1 preproprotein | ADAM18 | Q9Y3Q7 | 82.8 | 7.8 | 38.4 | 0.248 |
| gi\|142976675 | striatin-3 isoform 2 | STRN3 | Q13033 | 77.7 | 5 | 43.2 | 0.251 |
| gi\|4507725 | transthyretin precursor | TTR | P02766 | 15.9 | 5.4 | 246.3 | 0.252 |
| gi\|260064009 | ubiquitin carboxyl-terminal hydrolase 24 | USP24 | Q9UPU5 | 294.2 | 5.7 | 91.6 | 0.256 |
| gi\|154275767 | ADAMTS-like protein 1 isoform 4 precursor | ADAMTSL1 | Q8N6G6 | 193.3 | 9.5 | 69.2 | 0.256 |
| gi\|471270262 | otogelin precursor | OTOG | Q6ZRI0 | 314.6 | 5.5 | 114.0 | 0.265 |
| gi\|557129045 | mucin-2 precursor | MUC2 | Q02817 | 539.8 | 5.4 | 75.5 | 0.268 |
| gi\|28269707 | C-type lectin domain family 14 member A precursor | CLEC14A | Q86T13 | 51.6 | 6 | 40.3 | 0.273 |
| gi\|149588534 | ataxin-7-like protein 3 isoform b | ATXN7L3 | Q14CW9 | 38.6 | 6.8 | 36.6 | 0.278 |
| gi\|157384998 | low-density lipoprotein receptor-related protein 4 precursor | LRP4 | O75096 | 211.9 | 4.9 | 94.3 | 0.279 |
| gi\|332164775 | pyruvate kinase PKM isoform c | PKM | P14618 | 65.9 | 8.9 | 49.5 | 0.279 |
| gi\|394582140 | serine/threonine-protein phosphatase 2A 56 kDa regulatory subunit delta isoform isoform 4 | PPP2R5D | Q14738 | 53.1 | 6 | 41.8 | 0.286 |
| gi\|51093381 | leptin receptor isoform 2 precursor | LEPR | P48357 | 109.3 | 9 | 60.8 | 0.287 |
| gi\|308522758 | testis-specific Y-encoded protein 1 isoform TSPY-S | Q540R4 | Q540R4 | 33.1 | 5.3 | 37.7 | 0.287 |
| gi\|556503333 | nucleolar protein 9 isoform 2 | NOP9 | Q86U38 | 58.1 | 6.3 | 35.4 | 0.287 |
| gi\|150378539 | protein piccolo isoform 1 | PCLO | Q9Y6V0 | 560.4 | 6 | 156.2 | 0.288 |
| gi\|94966754 | elongation factor Tu GTP-binding domain-containing protein 1 isoform 1 | EFTUD1 | Q7Z2Z2 | 125.3 | 5.6 | 118.7 | 0.289 |
| gi\|557786188 | TBC1 domain family member 4 isoform 2 | TBC1D4 | O60343 | 145.6 | 6.7 | 65.4 | 0.291 |
| gi\|17738292 | D-beta-hydroxybutyrate dehydrogenase, mitochondrial precursor | BDH1 | Q02338 | 38.1 | 9.8 | 30.8 | 0.291 |
| gi\|153945734 | teashirt homolog 2 isoform 1 | TSHZ2 | Q9NRE2 | 114.9 | 8.9 | 66.2 | 0.292 |
| gi\|117956403 | rab GTPase-binding effector protein 2 | RABEP2 | Q9H5N1 | 63.5 | 4.6 | 36.5 | 0.292 |
| gi\|459683820 | NACHT, LRR and PYD domains-containing protein 12 isoform 4 | NLRP12 | P59046 | 113.8 | 6.8 | 61.6 | 0.296 |
| gi\|55769520 | inositol hexakisphosphate kinase 2 isoform a | IP6K2 | Q9UHH9 | 49.2 | 6.4 | 38.3 | 0.296 |
| gi\|6912622 | DNA repair and recombination protein RAD54B isoform 1 | RAD54B | Q9Y620 | 102.9 | 9.4 | 98.7 | 0.300 |
| gi\|31543060 | 4-hydroxy-2-oxoglutarate aldolase, mitochondrial isoform 1 | HOGA1 | Q86XE5 | 35.2 | 9.2 | 46.0 | 0.300 |
| gi\|350529405 | nuclear factor 1 C-type isoform 4 | NFIC | P08651 | 47.9 | 9.4 | 34.6 | 0.300 |
| gi\|259906018 | apoptotic chromatin condensation inducer in the nucleus isoform 2 | ACIN1 | Q9UKV3 | 150.5 | 6 | 32.8 | 0.300 |
| gi\|110349721 | titin isoform novex-3 | TTN | Q8WZ42 | 631.2 | 5.4 | 296.4 | 0.302 |
| gi\|34147544 | zinc finger protein 689 | ZNF689 | Q96CS4 | 56.9 | 11.7 | 58.9 | 0.304 |
| gi\|83700225 | potassium-transporting ATPase alpha chain 2 isoform 2 | ATP12A | P54707 | 115.4 | 6.1 | 45.4 | 0.309 |
| gi\|508083038 | F-box/LRR-repeat protein 7 isoform 2 | FBXL7 | Q9UJT9 | 49.8 | 10.2 | 30.8 | 0.310 |
| gi\|226529227 | zinc finger protein 716 | ZNF716 | A6NP11 | 57 | 10.3 | 61.7 | 0.313 |
| gi\|29294615 | taste receptor type 1 member 1 isoform b precursor | TAS1R1 | Q7RTX1 | 93 | 9.5 | 36.1 | 0.313 |
| gi\|55770834 | centromere protein F | CENPF | P49454 | 357.3 | 4.9 | 204.5 | 0.315 |
| gi\|188528648 | tenascin-X isoform 1 precursor | TNXB | P22105 | 457.9 | 4.9 | 142.6 | 0.315 |
| gi\|7705805 | 28S ribosomal protein S2, mitochondrial | MRPS2 | Q9Y399 | 33.2 | 10.1 | 29.9 | 0.321 |
| gi\|140560917 | myomesin-1 isoform b | MYOM1 | P52179 | 177.6 | 6.4 | 116.0 | 0.322 |
| gi\|62177129 | NEDD4-binding protein 3 | N4BP3 | O15049 | 60.4 | 9.3 | 82.9 | 0.330 |
| gi\|192807298 | voltage-dependent L-type calcium channel subunit alpha-1D isoform c | CACNA1D | Q01668 | 242.3 | 6.4 | 61.8 | 0.330 |
| gi\|299117128 | coronin-2B isoform 2 | CORO2B | Q9UQ03 | 54.4 | 9.3 | 43.9 | 0.331 |
| gi\|23308545 | transmembrane protein 74 | TMEM74 | Q96NL1 | 33.3 | 5 | 45.4 | 0.346 |
| gi\|256222411 | filamin-B isoform 1 | FLNB | O75369 | 281.5 | 5.4 | 120.4 | 0.348 |
| gi\|42415492 | mis18-binding protein 1 | MIS18BP1 | Q6P0N0 | 129 | 9.9 | 86.6 | 0.353 |
| gi\|157502191 | metastasis-associated in colon cancer protein 1 | MACC1 | Q6ZN28 | 96.6 | 6.5 | 44.4 | 0.357 |
| gi\|5032087 | splicing factor 3A subunit 1 isoform 1 | SF3A1 | Q15459 | 88.8 | 5 | 31.6 | 0.357 |
| gi\|122937273 | otolin-1 precursor | OTOL1 | A6NHN0 | 49.4 | 9.4 | 83.4 | 0.360 |
| gi\|4502511 | complement component C9 precursor | C9 | P02748 | 63.1 | 5.3 | 91.0 | 0.361 |
| gi\|108773808 | coiled-coil domain-containing protein 174 | CCDC174 | Q6PII3 | 53.9 | 6 | 84.6 | 0.361 |
| gi\|145275208 | myosin-IIIa | MYO3A | Q8NEV4 | 186.1 | 9.6 | 74.9 | 0.361 |
| gi\|156139139 | FAD-dependent oxidoreductase domain-containing protein 2 precursor | FOXRED2 | Q8IWF2 | 77.7 | 7.9 | 48.9 | 0.365 |
| gi\|226693363 | cytosolic phospholipase A2 gamma isoform 3 | PLA2G4C | Q9UP65 | 59.3 | 6.4 | 55.8 | 0.366 |
| gi\|223029420 | ski-like protein isoform 2 | SKIL | P12757 | 71.7 | 6.6 | 58.0 | 0.373 |
| gi\|38016937 | serine/threonine-protein kinase LMTK2 precursor | LMTK2 | Q8IWU2 | 164.8 | 4.2 | 43.9 | 0.376 |
| gi\|32401437 | testis-specific H1 histone | H1FNT | Q75WM6 | 28.1 | 12.4 | 38.6 | 0.380 |
| gi\|109150425 | ankyrin repeat and zinc finger domain-containing protein 1 isoform 1 | ANKZF1 | Q9H8Y5 | 80.9 | 9.6 | 46.3 | 0.383 |
| gi\|50659080 | alpha-1-antichymotrypsin precursor | SERPINA3 | P01011 | 47.6 | 5.2 | 279.4 | 0.386 |
| gi\|4507145 | sorting nexin-4 | SNX4 | O95219 | 51.9 | 5.6 | 43.3 | 0.398 |
| gi\|4826762 | haptoglobin isoform 1 preproprotein | HP | P00738 | 45.2 | 6.1 | 1449.9 | 0.402 |
| gi\|578836493 | PREDICTED: MORC family CW-type zinc finger protein 3 isoform X1 | B4DHJ4 | B4DHJ4 | 99.2 | 5.3 | 103.5 | 0.402 |
| gi\|533112494 | mucin-19 precursor | MUC19 | G3CIG0 | 804.8 | 4.8 | 93.9 | 0.402 |
| gi\|53759122 | adenomatous polyposis coli protein isoform b | APC | P25054 | 311.5 | 8.7 | 145.9 | 0.408 |
| gi\|530387865 | PREDICTED: histone-lysine N-methyltransferase NSD3 isoform X2 | WHSC1L1 | Q9BZ95 | 156 | 9.6 | 129.5 | 0.408 |
| gi\|325651904 | zinc finger protein 587B | ZNF587B | E7ETH6 | 45.5 | 9.8 | 43.8 | 0.408 |
| gi\|568786300 | zinc finger protein 286A isoform 1 | ZNF286A | Q9HBT8 | 65 | 9.5 | 64.3 | 0.410 |
| gi\|222537737 | ER degradation-enhancing alpha-mannosidase-like protein 2 isoform 1 precursor | EDEM2 | Q9BV94 | 64.7 | 5 | 37.1 | 0.410 |
| gi\|116284394 | myosin-14 isoform 2 | MYH14 | Q7Z406 | 227.7 | 5.4 | 115.0 | 0.417 |
| gi\|22550104 | ubiquitin carboxyl-terminal hydrolase 32 | USP32 | Q8NFA0 | 181.5 | 6 | 90.8 | 0.418 |
| gi\|40805852 | thrombospondin type-1 domain-containing protein 1 isoform 2 precursor | THSD1 | Q9NS62 | 88.7 | 9.5 | 63.1 | 0.418 |
| gi\|28605145 | glutamate receptor ionotropic, kainate 3 precursor | GRIK3 | Q13003 | 104 | 7.8 | 55.2 | 0.418 |
| gi\|530339650 | CUB and sushi domain-containing protein 2 isoform 1 | CSMD2 | Q7Z408 | 394.7 | 5.8 | 62.6 | 0.420 |
| gi\|568599847 | ephrin type-A receptor 7 isoform 2 precursor | EPHA7 | Q15375 | 111.4 | 5.5 | 55.9 | 0.420 |
| gi\|123173757 | ribonucleoprotein PTB-binding 1 | RAVER1 | Q8IY67 | 79.5 | 9.8 | 52.0 | 0.420 |
| gi\|157502212 | N-acetylgalactosaminyltransferase 7 | GALNT7 | Q86SF2 | 75.3 | 6.7 | 57.8 | 0.426 |
| gi\|195976777 | dysferlin isoform 3 | DYSF | O75923 | 235.8 | 5.5 | 85.2 | 0.427 |
| gi\|65787454 | apoptosis-inducing factor 3 isoform 2 | AIFM3 | Q96NN9 | 65.9 | 9.9 | 44.0 | 0.429 |
| gi\|160948599 | integrator complex subunit 1 | INTS1 | Q8N201 | 244.1 | 5.7 | 140.2 | 0.433 |
| gi\|27597061 | E3 ubiquitin-protein ligase UBR2 isoform 1 | UBR2 | Q8IWV8 | 200.4 | 5.8 | 59.2 | 0.433 |
| gi\|118600961 | ral GTPase-activating protein subunit alpha-2 | RALGAPA2 | Q2PPJ7 | 210.6 | 5.7 | 57.5 | 0.433 |
| gi\|14670392 | tyrosine-protein kinase BAZ1B | BAZ1B | Q9UIG0 | 170.8 | 9.4 | 118.1 | 0.435 |
| gi\|10880975 | coiled-coil domain-containing protein 181 | CCDC181 | Q5TID7 | 59.9 | 5.6 | 84.0 | 0.439 |
| gi\|356461016 | gem-associated protein 5 isoform 2 | GEMIN5 | Q8TEQ6 | 168.4 | 6.2 | 73.6 | 0.439 |
| gi\|221316699 | maltase-glucoamylase, intestinal | MGAM | O43451 | 209.7 | 5.2 | 44.5 | 0.439 |
| gi\|390407643 | cAMP-regulated phosphoprotein 21 isoform 4 | ARPP21 | Q9UBL0 | 88.5 | 6.5 | 41.1 | 0.439 |
| gi\|118421085 | treslin | TICRR | Q7Z2Z1 | 210.7 | 9.8 | 52.5 | 0.441 |
| gi\|157388955 | schlafen family member 12 | SLFN12 | Q8IYM2 | 66.9 | 9.6 | 37.1 | 0.442 |
| gi\|148491080 | trinucleotide repeat-containing gene 6B protein isoform 2 | TNRC6B | Q9UPQ9 | 182.7 | 6.6 | 83.5 | 0.450 |
| gi\|338753408 | transcription factor IIIB 90 kDa subunit isoform 5 | BRF1 | Q92994 | 61.8 | 5 | 60.8 | 0.455 |
| gi\|41054846 | armadillo repeat-containing X-linked protein 5 | ARMCX5 | Q6P1M9 | 62.3 | 9.5 | 157.2 | 0.459 |
| gi\|261878452 | repulsive guidance molecule A isoform 3 | RGMA | Q96B86 | 49.3 | 7.9 | 47.7 | 0.461 |
| gi\|194306543 | oxidation resistance protein 1 isoform 2 | OXR1 | Q8N573 | 93.7 | 4.9 | 48.1 | 0.463 |
| gi\|221316711 | piwi-like protein 4 | PIWIL4 | Q7Z3Z4 | 96.5 | 9.9 | 41.4 | 0.465 |
| gi\|255759952 | WD repeat-containing protein 81 isoform 1 | WDR81 | Q562E7 | 211.6 | 5.3 | 124.2 | 0.472 |
| gi\|332688227 | dynein heavy chain 8, axonemal | DNAH8 | Q96JB1 | 538.3 | 5.7 | 211.7 | 0.474 |
| gi\|5453998 | importin-7 | IPO7 | O95373 | 119.4 | 4.6 | 91.6 | 0.474 |
| gi\|578832904 | PREDICTED: adenomatous polyposis coli protein 2 isoform X5 | APC2 | O95996 | 243.7 | 10 | 228.1 | 0.478 |
| gi\|21264565 | AT-rich interactive domain-containing protein 1A isoform a | ARID1A | O14497 | 241.9 | 6.2 | 95.7 | 0.478 |
| gi\|545687835 | DEP domain-containing mTOR-interacting protein isoform 2 | DEPTOR | Q8TB45 | 34 | 7.9 | 49.3 | 0.481 |
| gi\|222352127 | protein sidekick-2 precursor | SDK2 | Q58EX2 | 239.2 | 6.6 | 85.3 | 0.483 |
| gi\|324021730 | rho GTPase-activating protein 19 isoform 2 | ARHGAP19 | Q14CB8 | 52.4 | 10 | 76.5 | 0.483 |
| gi\|157676340 | zinc finger protein 142 | ZNF142 | P52746 | 187.8 | 9.5 | 63.1 | 0.483 |
| gi\|378925630 | ubiquitin carboxyl-terminal hydrolase 17-like protein 10 | USP17L10 | C9JJH3 | 59.8 | 9.4 | 50.5 | 0.483 |
| gi\|72534670 | phospholipase A-2-activating protein | PLAA | Q9Y263 | 87.1 | 5.9 | 38.7 | 0.483 |
| gi\|83367077 | mucin-16 | MUC16 | Q8WXI7 | 1518.2 | 5 | 220.9 | 0.490 |
| gi\|270265793 | stAR-related lipid transfer protein 9 | STARD9 | Q9P2P6 | 516 | 5.9 | 182.0 | 0.495 |
| gi\|58082081 | phosphoinositide 3-kinase regulatory subunit 6 | PIK3R6 | Q5UE93 | 84.2 | 8.6 | 112.7 | 0.495 |
| gi\|13899221 | gasdermin-C | GSDMC | Q9BYG8 | 57.7 | 5.5 | 64.4 | 0.495 |
| gi\|194248068 | ras/Rap GTPase-activating protein SynGAP | SYNGAP1 | Q96PV0 | 148.2 | 9.8 | 36.0 | 0.495 |
| gi\|27436940 | reelin isoform b precursor | RELN | P78509 | 388 | 5.5 | 74.0 | 0.498 |
| gi\|62198237 | DNA polymerase epsilon catalytic subunit A | POLE | Q07864 | 261.4 | 6 | 91.8 | 0.500 |
| gi\|140161498 | microtubule-associated tumor suppressor candidate 2 isoform a | MTUS2 | Q5JR59 | 151.1 | 6.3 | 82.3 | 0.503 |
| gi\|530377917 | PREDICTED: protein SDA1 homolog isoform X4 | SDAD1 | Q9NVU7 | 68.4 | 10 | 48.3 | 0.505 |
| gi\|126012562 | prolow-density lipoprotein receptor-related protein 1 precursor | LRP1 | Q07954 | 504.3 | 5 | 284.2 | 0.508 |
| gi\|40806160 | thyroid hormone receptor alpha isoform 1 | THRA | P10827 | 46.8 | 9 | 45.1 | 0.508 |
| gi\|117938328 | lethal(3)malignant brain tumor-like protein 1 isoform I | L3MBTL1 | Q9Y468 | 85.9 | 5.7 | 35.8 | 0.508 |
| gi\|21704279 | protein jagged-2 isoform b precursor | JAG2 | Q9Y219 | 129.1 | 5.4 | 94.6 | 0.510 |
| gi\|281604114 | FERM and PDZ domain-containing protein 2 isoform 3 | FRMPD2 | Q68DX3 | 144.2 | 6.3 | 92.4 | 0.510 |
| gi\|89886453 | TBC1 domain family member 24 isoform 2 | TBC1D24 | Q9ULP9 | 62.3 | 7.1 | 42.1 | 0.510 |
| gi\|52630322 | chromodomain-helicase-DNA-binding protein 3 isoform 2 | CHD3 | Q12873 | 222.7 | 7.6 | 147.5 | 0.518 |
| gi\|23510366 | homeobox protein Hox-D10 | HOXD10 | P28358 | 38.4 | 9.5 | 42.0 | 0.518 |
| gi\|255759908 | putative helicase Mov10l1 isoform 3 | MOV10L1 | Q9BXT6 | 130.1 | 8.6 | 93.6 | 0.521 |
| gi\|4504125 | glutamate receptor ionotropic, NMDA 2A isoform 1 precursor | GRIN2A | Q12879 | 165.2 | 6.7 | 43.6 | 0.521 |
| gi\|119120907 | partitioning defective 3 homolog B isoform a | PARD3B | Q8TEW8 | 121.1 | 9.4 | 41.7 | 0.521 |
| gi\|388596709 | sorting nexin-17 isoform 4 | B4DQ37 | B4DQ37 | 50.8 | 9 | 55.3 | 0.524 |
| gi\|7661864 | USP6 N-terminal-like protein isoform 1 | USP6NL | Q92738 | 94 | 9.5 | 41.2 | 0.524 |
| gi\|226442729 | kelch-like protein 15 | KLHL15 | Q96M94 | 69.7 | 6 | 31.0 | 0.524 |
| gi\|578831636 | PREDICTED: peripheral-type benzodiazepine receptor-associated protein 1 isoform X2 | BZRAP1 | O95153 | 204.9 | 4.9 | 112.2 | 0.529 |
| gi\|144446030 | lymphocyte antigen 75 precursor | LY75 | O60449 | 198.2 | 6.2 | 54.1 | 0.529 |
| gi\|166235184 | paladin | PALD1 | Q9ULE6 | 96.7 | 6.1 | 58.5 | 0.532 |
| gi\|32967603 | bromodomain adjacent to zinc finger domain protein 1A isoform a | BAZ1A | Q9NRL2 | 178.6 | 6.2 | 59.6 | 0.538 |
| gi\|321267571 | CASP8 and FADD-like apoptosis regulator isoform 6 | CFLAR | O15519 | 41.3 | 7 | 51.9 | 0.538 |
| gi\|231573214 | E3 ubiquitin-protein ligase listerin | LTN1 | O94822 | 205 | 6.1 | 81.5 | 1.961 |
| gi\|55953135 | immunoglobulin superfamily member 3 isoform 2 precursor | IGSF3 | O75054 | 135.1 | 5.7 | 79.9 | 1.961 |
| gi\|31317307 | proprotein convertase subtilisin/kexin type 9 preproprotein | PCSK9 | Q8NBP7 | 74.2 | 6.1 | 70.3 | 1.961 |
| gi\|530397034 | PREDICTED: N-terminal kinase-like protein isoform X3 | SCYL1 | Q96KG9 | 86.3 | 6.1 | 66.7 | 1.961 |
| gi\|356582335 | serine/threonine-protein kinase PLK2 isoform 2 | PLK2 | Q9NYY3 | 76.6 | 9.4 | 62.1 | 1.961 |
| gi\|359806543 | alpha-protein kinase 1 isoform 2 | ALPK1 | Q96QP1 | 129.9 | 6.1 | 44.5 | 1.961 |
| gi\|262118282 | plexin-A1 precursor | PLXNA1 | Q9UIW2 | 210.9 | 6.5 | 38.5 | 1.961 |
| gi\|7706657 | pre-mRNA-processing factor 17 | CDC40 | O60508 | 65.5 | 6.7 | 33.0 | 1.961 |
| gi\|544583520 | cyclin-Y isoform 3 | CCNY | Q8ND76 | 36.5 | 8.7 | 30.8 | 1.961 |
| gi\|4557871 | serotransferrin precursor | TF | P02787 | 77 | 7 | 3340.6 | 2.000 |
| gi\|238859593 | UDP-glucose:glycoprotein glucosyltransferase 2 precursor | UGGT2 | Q9NYU1 | 174.6 | 6.4 | 91.6 | 2.000 |
| gi\|12667788 | myosin-9 | MYH9 | P35579 | 226.4 | 5.4 | 214.1 | 2.041 |
| gi\|4501987 | afamin precursor | AFM | P43652 | 69 | 5.6 | 180.8 | 2.041 |
| gi\|151301127 | dynein heavy chain 7, axonemal | DNAH7 | Q8WXX0 | 460.9 | 5.6 | 155.1 | 2.041 |
| gi\|530396906 | PREDICTED: synaptotagmin-like protein 2 isoform X1 | SYTL2 | A0A0U1RR07 | 247.2 | 5.8 | 154.2 | 2.041 |
| gi\|574281382 | slit homolog 2 protein isoform 3 precursor | SLIT2 | O94813 | 168.8 | 6.9 | 101.1 | 2.041 |
| gi\|392050772 | zinc finger protein 850 isoform 2 | ZNF850 | A0A087X0M6 | 121.8 | 10.1 | 37.5 | 2.041 |
| gi\|55953087 | nucleolar GTP-binding protein 1 | GTPBP4 | Q9BZE4 | 73.9 | 10 | 102.5 | 2.083 |
| gi\|530388147 | PREDICTED: LON peptidase N-terminal domain and RING finger protein 1 isoform X1 | LONRF1 | Q17RB8 | 85.4 | 8.5 | 87.8 | 2.083 |
| gi\|4502157 | apolipoprotein C-I precursor | APOC1 | P02654 | 9.3 | 9.3 | 73.5 | 2.083 |
| gi\|46049119 | myosin-binding protein C, slow-type isoform 3 | MYBPC1 | Q00872 | 128.2 | 5.7 | 58.8 | 2.083 |
| gi\|544063391 | protein kinase C theta type isoform 4 | PRKCQ | Q04759 | 67.5 | 8.6 | 33.6 | 2.083 |
| gi\|122937211 | proteasome-associated protein ECM29 homolog | KIAA0368 | J3KN16 | 223.6 | 9.7 | 81.5 | 2.128 |
| gi\|22749429 | coiled-coil domain-containing protein 89 | CCDC89 | Q8N998 | 43.8 | 5.2 | 38.2 | 2.128 |
| gi\|4507955 | transcriptional repressor protein YY1 | YY1 | P25490 | 44.7 | 5.8 | 38.1 | 2.128 |
| gi\|70778918 | inter-alpha-trypsin inhibitor heavy chain H2 precursor | ITIH2 | P19823 | 106.4 | 6.4 | 373.2 | 2.174 |
| gi\|19913408 | DNA topoisomerase 2-beta | TOP2B | Q02880 | 182.5 | 8.9 | 152.4 | 2.174 |
| gi\|63998985 | mitogen-activated protein kinase kinase kinase 19 isoform 3 | MAP3K19 | Q56UN5 | 137.5 | 6.7 | 123.6 | 2.174 |
| gi\|33620769 | E3 ubiquitin-protein ligase RBBP6 isoform 1 | RBBP6 | Q7Z6E9 | 201.4 | 10.2 | 89.3 | 2.174 |
| gi\|82546830 | exocyst complex component 4 isoform a | EXOC4 | Q96A65 | 110.4 | 6.1 | 82.0 | 2.174 |
| gi\|14150185 | zinc finger BED domain-containing protein 3 | ZBED3 | Q96IU2 | 25.1 | 10.3 | 34.8 | 2.174 |
| gi\|578805674 | PREDICTED: catenin beta-1 isoform X2 | CTNNB1 | B4DGU4 | 84.7 | 5.5 | 79.2 | 2.222 |
| gi\|22035692 | GDNF family receptor alpha-1 isoform b preproprotein | GFRA1 | P56159 | 50.8 | 9.6 | 67.6 | 2.222 |
| gi\|557948073 | synaptopodin-2 isoform d | SYNPO2 | Q9UMS6 | 133 | 9.5 | 57.9 | 2.222 |
| gi\|257796256 | aminomethyltransferase, mitochondrial isoform 3 precursor | AMT | P48728 | 37.4 | 9.9 | 41.5 | 2.222 |
| gi\|56605998 | EP300-interacting inhibitor of differentiation 3 | EID3 | Q8N140 | 38.1 | 4.9 | 32.4 | 2.222 |
| gi\|262118216 | coiled-coil domain-containing protein 88B precursor | CCDC88B | A6NC98 | 164.7 | 4.9 | 83.1 | 2.273 |
| gi\|530402206 | PREDICTED: alpha-tectorin isoform X1 | ZC3H13 | Q5T200 | 238.8 | 5.1 | 124.1 | 2.326 |
| gi\|4507879 | voltage-dependent anion-selective channel protein 1 | VDAC1 | P21796 | 30.8 | 9.2 | 54.2 | 2.326 |
| gi\|122937345 | unconventional myosin-Vb | MYO5B | Q9ULV0 | 213.5 | 6.8 | 49.9 | 2.326 |
| gi\|284925165 | SUN domain-containing protein 1 isoform c | SUN1 | O94901 | 76.4 | 6.2 | 40.4 | 2.326 |
| gi\|542133148 | ubiquitin-associated protein 2 isoform 1 | UBAP2 | Q5T6F2 | 117.1 | 7 | 31.2 | 2.326 |
| gi\|4502027 | serum albumin preproprotein | ALB | P02768 | 69.3 | 5.9 | 738.9 | 2.381 |
| gi\|189083780 | gelsolin isoform c | GSN | P06396 | 81.9 | 5.5 | 146.0 | 2.381 |
| gi\|4507241 | FACT complex subunit SSRP1 | SSRP1 | Q08945 | 81 | 6.5 | 60.4 | 2.381 |
| gi\|11415040 | suppressor of tumorigenicity 14 protein | ST14 | Q9Y5Y6 | 94.7 | 6.1 | 57.3 | 2.381 |
| gi\|289547536 | Fanconi anemia-associated protein of 100 kDa isoform b | FAAP100 | Q0VG06 | 93.4 | 5 | 38.5 | 2.381 |
| gi\|5032315 | dystrophin Dp427p2 isoform | DMD | P11532 | 412.1 | 5.5 | 234.8 | 2.439 |
| gi\|55770888 | early endosome antigen 1 | EEA1 | Q15075 | 162.4 | 5.4 | 76.9 | 2.439 |
| gi\|91208423 | thyroid receptor-interacting protein 6 | TRIP6 | Q15654 | 50.3 | 8 | 30.5 | 2.439 |
| gi\|299829223 | coiled-coil domain-containing protein 141 | CCDC141 | Q6ZP82 | 175 | 5.5 | 147.3 | 2.500 |
| gi\|148833508 | neurogenic locus notch homolog protein 1 preproprotein | NOTCH1 | P46531 | 272.3 | 4.8 | 107.2 | 2.500 |
| gi\|157671949 | zinc finger protein Rlf | RLF | Q13129 | 217.8 | 6.3 | 90.0 | 2.500 |
| gi\|156139127 | uncharacterized protein C10orf88 | C10orf88 | Q9H8K7 | 49.2 | 5.8 | 57.7 | 2.500 |
| gi\|6912464 | latrophilin-2 precursor | ADGRL2 | O95490 | 157.1 | 5.8 | 34.4 | 2.500 |
| gi\|143770880 | laminin subunit beta-4 precursor | LAMB4 | A4D0S4 | 193.4 | 5.9 | 150.5 | 2.564 |
| gi\|40254834 | kinesin-like protein KIF1C | KIF1C | O43896 | 122.9 | 6.5 | 125.8 | 2.564 |
| gi\|31083193 | adenylate cyclase type 1 isoform 1 | ADCY1 | Q08828 | 123.4 | 9.7 | 85.1 | 2.564 |
| gi\|157266264 | pleckstrin homology-like domain family B member 3 | PHLDB3 | Q6NSJ2 | 71.9 | 6.1 | 80.1 | 2.564 |
| gi\|4502365 | homeobox protein Nkx-3.2 | NKX3-2 | P78367 | 34.8 | 9.2 | 58.4 | 2.564 |
| gi\|83035129 | coiled-coil domain-containing protein 108 isoform 1 | CCDC108 | Q6ZU64 | 217.1 | 6.1 | 69.5 | 2.632 |
| gi\|4758766 | bifunctional heparan sulfate N-deacetylase/N-sulfotransferase 3 | NDST3 | O95803 | 100.8 | 8.9 | 53.2 | 2.632 |
| gi\|70610136 | poly(ADP-ribose) glycohydrolase | PARG | Q86W56 | 111 | 6 | 51.0 | 2.632 |
| gi\|57634534 | nuclear pore complex protein Nup205 | NUP205 | Q92621 | 227.8 | 5.8 | 75.2 | 2.778 |
| gi\|6912724 | tolloid-like protein 2 precursor | TLL2 | Q9Y6L7 | 113.5 | 5.5 | 67.9 | 2.778 |
| gi\|157427661 | APC membrane recruitment protein 3 | AMER3 | Q8N944 | 90.4 | 5.4 | 60.4 | 2.778 |
| gi\|118150678 | epithelial cell-transforming sequence 2 oncogene-like | ECT2L | Q008S8 | 104.8 | 9.4 | 52.9 | 2.778 |
| gi\|4758012 | clathrin heavy chain 1 isoform 1 | CLTC | Q00610 | 191.5 | 5.4 | 43.9 | 2.778 |
| gi\|298286489 | putative adenosylhomocysteinase 3 isoform c | AHCYL2 | Q96HN2 | 56.7 | 9.4 | 36.3 | 2.778 |
| gi\|156523970 | alpha-2-HS-glycoprotein preproprotein | AHSG | P02765 | 39.3 | 5.4 | 439.2 | 2.857 |
| gi\|298676524 | zinc finger protein 674 isoform 3 | ZNF674 | Q2M3X9 | 66.6 | 10.2 | 115.4 | 2.857 |
| gi\|268607667 | epidermal growth factor-like protein 6 isoform 2 precursor | EGFL6 | Q8IUX8 | 61.3 | 9.7 | 82.8 | 2.857 |
| gi\|530397573 | PREDICTED: synaptotagmin-7 isoform X3 | SYT7 | F5H1N2 | 68.4 | 9.9 | 66.3 | 2.857 |
| gi\|4885583 | rho-associated protein kinase 1 [Homo sapiens] | ROCK1 | Q13464 | 158.1 | 5.6 | 113.3 | 2.941 |
| gi\|114145479 | neuroepithelial cell-transforming gene 1 protein isoform 1 | NET1 | Q7Z628 | 67.7 | 10 | 107.7 | 2.941 |
| gi\|44921615 | exocyst complex component 8 | EXOC8 | Q8IYI6 | 81.7 | 5.2 | 78.1 | 2.941 |
| gi\|149363685 | uncharacterized protein KIAA0947 | ICE1 | Q9Y2F5 | 247.7 | 5.2 | 46.6 | 2.941 |
| gi\|156616273 | pleckstrin | PLEK | P08567 | 40.1 | 9.3 | 37.1 | 2.941 |
| gi\|90903231 | huntingtin | HTT | P42858 | 347.6 | 5.8 | 102.1 | 3.030 |
| gi\|495528154 | collagen alpha-1(V) chain isoform 2 preproprotein | COL5A1 | A0A087WXW9 | 183.5 | 4.8 | 92.9 | 3.030 |
| gi\|28626504 | fermitin family homolog 3 short form | FERMT3 | Q86UX7 | 75.4 | 6.3 | 49.5 | 3.030 |
| gi\|11415038 | solute carrier family 22 member 3 | SLC22A3 | O75751 | 61.2 | 10 | 42.0 | 3.030 |
| gi\|33624896 | A disintegrin and metalloproteinase with thrombospondin motifs 9 preproprotein | ADAMTS9 | Q9P2N4 | 216.4 | 9.4 | 134.3 | 3.125 |
| gi\|170016077 | probable cation-transporting ATPase 13A1 | ATP13A1 | Q9HD20 | 132.9 | 9.4 | 81.7 | 3.125 |
| gi\|21361912 | dnaJ homolog subfamily C member 1 precursor | DNAJC1 | Q96KC8 | 63.8 | 9.4 | 81.5 | 3.125 |
| gi\|119829187 | collagen alpha-1(XVII) chain | COL17A1 | Q9UMD9 | 150.3 | 9.4 | 80.8 | 3.125 |
| gi\|74315348 | transient receptor potential cation channel subfamily V member 1 | TRPV1 | Q8NER1 | 94.9 | 7.1 | 66.7 | 3.125 |
| gi\|38201692 | ras GTPase-activating protein 3 | RASA3 | Q14644 | 95.6 | 6.8 | 56.2 | 3.125 |
| gi\|62177127 | unconventional myosin-XVI isoform 2 | MYO16 | Q9Y6X6 | 206 | 6.4 | 52.8 | 3.125 |
| gi\|195927059 | axin-2 | AXIN2 | Q9Y2T1 | 93.5 | 8.7 | 38.0 | 3.125 |
| gi\|315113881 | receptor-type tyrosine-protein phosphatase-like N isoform 3 | PTPRN | Q16849 | 96.2 | 6.1 | 48.2 | 3.226 |
| gi\|27477109 | E3 ubiquitin-protein ligase Itchy homolog isoform 2 | ITCH | Q96J02 | 98.6 | 5.9 | 85.7 | 3.333 |
| gi\|7669528 | pro-neuregulin-2, membrane-bound isoform isoform 2 | NRG2 | O14511 | 90.8 | 10.4 | 59.4 | 3.333 |
| gi\|312283633 | tudor domain-containing protein 5 isoform 1 | TDRD5 | Q8NAT2 | 116 | 7.3 | 51.0 | 3.333 |
| gi\|262527235 | lysosomal protective protein isoform c precursor | CTSA | P10619 | 54.2 | 6.6 | 74.2 | 3.448 |
| gi\|21361337 | eukaryotic translation initiation factor 5 | EIF5 | P55010 | 49.2 | 5.3 | 62.4 | 3.704 |
| gi\|118402596 | cell division cycle protein 23 homolog | CDC23 | Q9UJX2 | 68.8 | 6.6 | 61.9 | 3.704 |
| gi\|38045956 | zinc finger protein 19 | ZNF19 | P17023 | 52.4 | 9.1 | 52.1 | 3.704 |
| gi\|48717241 | zinc finger protein 425 | ZNF425 | Q6IV72 | 87.7 | 10.6 | 49.7 | 3.704 |
| gi\|229892308 | shugoshin-like 2 isoform 2 | B7Z7S9 | B7Z7S9 | 143.9 | 8.5 | 46.2 | 3.704 |
| gi\|134133226 | POTE ankyrin domain family member E | POTEE | Q6S8J3 | 121.3 | 5.8 | 102.6 | 3.846 |
| gi\|282847476 | peptidyl-prolyl cis-trans isomerase NIMA-interacting 4 isoform 2 | PIN4 | Q9Y237 | 14.2 | 10.7 | 73.2 | 3.846 |
| gi\|34304360 | nephrocystin-3 | NPHP3 | Q7Z494 | 150.8 | 6.3 | 112.0 | 4.000 |
| gi\|115430237 | spectrin beta chain, non-erythrocytic 4 isoform sigma1 | SPTBN4 | Q9H254 | 288.8 | 5.7 | 114.1 | 4.167 |
| gi\|66730421 | probable cation-transporting ATPase 13A5 | ATP13A5 | Q4VNC0 | 137.2 | 9.1 | 58.4 | 4.167 |
| gi\|289547591 | 1-phosphatidylinositol 4,5-bisphosphate phosphodiesterase beta-4 isoform b | PLCB4 | Q15147 | 134.4 | 6.5 | 57.4 | 4.167 |
| gi\|31657123 | Hermansky-Pudlak syndrome 5 protein isoform a | HPS5 | Q9UPZ3 | 127.4 | 5.2 | 36.5 | 4.167 |
| gi\|183603931 | serine/threonine-protein phosphatase 6 catalytic subunit isoform c | PPP6C | O00743 | 32.5 | 5.6 | 69.5 | 4.762 |
| gi\|33620745 | pre-mRNA cleavage complex 2 protein Pcf11 | PCF11 | O94913 | 172.9 | 9.3 | 78.2 | 5.556 |
| gi\|189217897 | syntaxin-binding protein 5 isoform a | STXBP5 | Q5T5C0 | 123.4 | 6.8 | 56.1 | 5.882 |
| gi\|38570117 | zinc finger protein 569 | ZNF569 | Q5MCW4 | 79.5 | 10 | 54.4 | 5.882 |
| gi\|147905620 | histone-lysine N-methyltransferase PRDM9 | PRDM9 | Q9NQV7 | 103.3 | 10.3 | 45.6 | 5.882 |
| gi\|19923345 | RNA-binding protein 12 | RBM12 | Q9NTZ6 | 97.3 | 9.4 | 43.5 | 5.882 |
| gi\|31742484 | erythroid differentiation-related factor 1 isoform 2 | EDRF1 | Q3B7T1 | 134.8 | 6 | 95.2 | 7.143 |
| gi\|459642365 | dynein heavy chain 11, axonemal | DNAH11 | Q96DT5 | 520 | 6 | 140.3 | 8.333 |
| gi\|304766736 | proline dehydrogenase 1, mitochondrial isoform 1 precursor | PRODH | O43272 | 67.9 | 8.8 | 79.3 | 16.667 |
