# Supplementary material for: Screening and Identification of APOC1 as a Novel Potential Biomarker for Differentiate of Mycoplasma pneumoniae in Children
Source: Front Microbiol. 2016 Dec 15;7:1961. doi: 10.3389/fmicb.2016.01961 (PMC5156883; doi:10.3389/fmicb.2016.01961)
Supplement: Supplementary file 3 [file Table3.DOCX]

**Supplementary 3: Proteins identified following LC-MS/MS of MPP different from disease control fraction.**

| Gi number | Protein name | Gene | Uniprot Identifier | Mass | pI | Scores | MPP:IDC |
| --- | --- | --- | --- | --- | --- | --- | --- |
| gi\|302129652 | annexin A6 isoform 2 | ANXA6 | P08133 | 72.4 | 5.3 | 71.3 | 0.103 |
| gi\|4507955 | transcriptional repressor protein YY1 | YY1 | P25490 | 44.7 | 5.8 | 38.1 | 0.124 |
| gi\|40549448 | guanine nucleotide exchange factor VAV2 isoform 2 | VAV2 | P52735 | 97 | 6.5 | 45.3 | 0.139 |
| gi\|31791053 | zinc finger protein 804B | ZNF804B | A4D1E1 | 152.5 | 9.8 | 116.8 | 0.189 |
| gi\|183583553 | collagen alpha-5(VI) chain isoform 2 precursor | COL6A5 | A8TX70 | 279.8 | 7.1 | 120.5 | 0.192 |
| gi\|16418425 | guanylate-binding protein 5 | GBP5 | Q96PP8 | 66.6 | 5.3 | 55.7 | 0.192 |
| gi\|557947982 | spermatogenesis-associated protein 13 isoform 5 | SPATA13 | Q96N96 | 69.1 | 7.2 | 48.7 | 0.192 |
| gi\|566559824 | CAP-Gly domain-containing linker protein 4 isoform 2 | CLIP4 | Q8N3C7 | 64.9 | 9.5 | 45.9 | 0.192 |
| gi\|21237808 | SWI/SNF complex subunit SMARCC2 isoform b | SMARCC2 | Q8TAQ2 | 124.8 | 5.3 | 32.8 | 0.192 |
| gi\|83716024 | kinesin-like protein KIF21B isoform 2 | KIF21B | O75037 | 181.2 | 7.2 | 47.7 | 0.220 |
| gi\|32967603 | bromodomain adjacent to zinc finger domain protein 1A isoform a | BAZ1A | Q9NRL2 | 178.6 | 6.2 | 59.6 | 0.227 |
| gi\|270265812 | mitochondrial cardiolipin hydrolase | PLD6 | Q8N2A8 | 28.3 | 10.8 | 30.2 | 0.236 |
| gi\|7661960 | kinetochore-associated protein 1 | KNTC1 | P50748 | 250.6 | 5.6 | 127.2 | 0.264 |
| gi\|108773808 | coiled-coil domain-containing protein 174 | CCDC174 | Q6PII3 | 53.9 | 6 | 84.6 | 0.266 |
| gi\|578798757 | PREDICTED: protein SZT2 isoform X2 | SZT2 | Q5T011 | 377.8 | 5.8 | 148.0 | 0.269 |
| gi\|58532587 | BTB/POZ domain-containing protein 7 isoform 1 | BTBD7 | Q9P203 | 126.3 | 6.5 | 39.2 | 0.271 |
| gi\|338797736 | protein phosphatase 1 regulatory subunit 37 | PPP1R37 | O75864 | 74.7 | 4.8 | 32.6 | 0.271 |
| gi\|530372926 | PREDICTED: RAF proto-oncogene serine/threonine-protein kinase isoform X3 | RAF1 | A0A0S2Z4L5 | 69.5 | 10.1 | 74.3 | 0.276 |
| gi\|29725624 | collagen alpha-1(XXIII) chain | COL23A1 | Q86Y22 | 51.9 | 7.7 | 63.8 | 0.276 |
| gi\|66346695 | fibrillin-2 precursor | FBN2 | P35556 | 314.6 | 4.6 | 172.9 | 0.310 |
| gi\|190684646 | RAS guanyl-releasing protein 1 isoform b | RASGRP1 | O95267 | 86.6 | 8.9 | 50.4 | 0.313 |
| gi\|262118216 | coiled-coil domain-containing protein 88B precursor | CCDC88B | A6NC98 | 164.7 | 4.9 | 83.1 | 0.328 |
| gi\|21626468 | zinc finger protein 638 isoform 1 | ZNF638 | Q14966 | 220.5 | 6 | 105.4 | 0.337 |
| gi\|282402177 | cyclin-dependent kinase 20 isoform 5 | CDK20 | Q8IZL9 | 27.1 | 11 | 59.6 | 0.369 |
| gi\|338753408 | transcription factor IIIB 90 kDa subunit isoform 5 | BRF1 | Q92994 | 61.8 | 5 | 60.8 | 0.372 |
| gi\|293597572 | transient receptor potential cation channel subfamily M member 6 isoform b | TRPM6 | Q9BX84 | 230.9 | 8.7 | 115.9 | 0.376 |
| gi\|14141170 | metastasis-associated protein MTA2 | MTA2 | O94776 | 75 | 10.4 | 39.8 | 0.380 |
| gi\|149588534 | ataxin-7-like protein 3 isoform b | ATXN7L3 | Q14CW9 | 38.6 | 6.8 | 36.6 | 0.407 |
| gi\|282165714 | transmembrane protein 132C precursor | TMEM132C | Q8N3T6 | 121.7 | 5.9 | 61.1 | 0.408 |
| gi\|4507987 | zinc finger protein 136 | ZNF136 | P52737 | 62.7 | 10.3 | 61.7 | 0.412 |
| gi\|221219000 | inactive phospholipase C-like protein 2 isoform 1 | PLCL2 | Q9UPR0 | 125.8 | 6.5 | 49.9 | 0.412 |
| gi\|390407643 | cAMP-regulated phosphoprotein 21 isoform 4 | ARPP21 | Q9UBL0 | 88.5 | 6.5 | 41.1 | 0.422 |
| gi\|4885583 | rho-associated protein kinase 1 | ROCK1 | Q13464 | 158.1 | 5.6 | 113.3 | 0.431 |
| gi\|44921615 | exocyst complex component 8 | EXOC8 | Q8IYI6 | 81.7 | 5.2 | 78.1 | 0.431 |
| gi\|93204879 | PR domain zinc finger protein 15 isoform 1 | PRDM15 | P57071 | 169.2 | 9.6 | 123.4 | 0.439 |
| gi\|38201625 | eukaryotic translation initiation factor 4 gamma 1 isoform 3 | EIF4G1 | Q04637 | 158.4 | 5 | 58.2 | 0.439 |
| gi\|530425693 | PREDICTED: zinc finger protein 337 isoform X1 | ZNF337 | Q9Y3M9 | 83.1 | 10.9 | 45.7 | 0.439 |
| gi\|83700225 | potassium-transporting ATPase alpha chain 2 isoform 2 | ATP12A | P54707 | 115.4 | 6.1 | 45.4 | 0.439 |
| gi\|47419909 | transcription intermediary factor 1-alpha isoform b | TRIM24 | O15164 | 112.9 | 6.4 | 65.2 | 0.448 |
| gi\|269973871 | kelch-like protein 13 isoform e | KLHL13 | Q9P2N7 | 69 | 6 | 53.2 | 0.448 |
| gi\|4557321 | apolipoprotein A-I preproprotein | APOA1 | P02647 | 30.8 | 5.5 | 1487.5 | 0.461 |
| gi\|154091334 | protein MRVI1 isoform c | MRVI1 | Q9Y6F6 | 65.8 | 5.2 | 72.7 | 0.476 |
| gi\|296317239 | peroxisomal membrane protein 11B isoform 2 | PEX11B | O96011 | 26.7 | 11.1 | 44.9 | 0.478 |
| gi\|50659080 | alpha-1-antichymotrypsin precursor | SERPINA3 | P01011 | 47.6 | 5.2 | 279.4 | 0.483 |
| gi\|321267571 | CASP8 and FADD-like apoptosis regulator isoform 6 | CFLAR | O15519 | 41.3 | 7 | 51.9 | 0.493 |
| gi\|74099694 | sulfite oxidase, mitochondrial | SUOX | P51687 | 60.2 | 5.7 | 48.3 | 0.500 |
| gi\|187761343 | acyl-CoA synthetase family member 3, mitochondrial isoform 1 precursor | ACSF3 | Q4G176 | 64.1 | 9.5 | 66.4 | 0.503 |
| gi\|31415870 | dedicator of cytokinesis protein 3 | DOCK3 | Q8IZD9 | 233 | 6.5 | 56.3 | 0.513 |
| gi\|140161498 | microtubule-associated tumor suppressor candidate 2 isoform a | MTUS2 | Q5JR59 | 151.1 | 6.3 | 82.3 | 0.526 |
| gi\|356461016 | gem-associated protein 5 isoform 2 | GEMIN5 | Q8TEQ6 | 168.4 | 6.2 | 73.6 | 0.526 |
| gi\|33620769 | E3 ubiquitin-protein ligase RBBP6 isoform 1 | RBBP6 | Q7Z6E9 | 201.4 | 10.2 | 89.3 | 0.529 |
| gi\|431822375 | dedicator of cytokinesis protein 7 isoform 1 | DOCK7 | Q96N67 | 241.3 | 6.4 | 91.4 | 0.535 |
| gi\|24497531 | ellis-van Creveld syndrome protein | EVC | P57679 | 111.9 | 6.3 | 49.5 | 0.538 |
| gi\|222352127 | protein sidekick-2 precursor | SDK2 | Q58EX2 | 239.2 | 6.6 | 85.3 | 0.546 |
| gi\|378925630 | ubiquitin carboxyl-terminal hydrolase 17-like protein 10 | USP17L10 | C9JJH3 | 59.8 | 9.4 | 50.5 | 0.546 |
| gi\|63998985 | mitogen-activated protein kinase kinase kinase 19 isoform 3 | MAP3K19 | Q56UN5 | 137.5 | 6.7 | 123.6 | 1.961 |
| gi\|346986273 | rho guanine nucleotide exchange factor 28 isoform 3 | ARHGEF28 | Q8N1W1 | 157.2 | 6 | 55.8 | 1.961 |
| gi\|46411172 | muscleblind-like protein 1 isoform f | MBNL1 | Q9NR56 | 37 | 10.2 | 46.5 | 1.961 |
| gi\|41406086 | semaphorin-3D precursor | SEMA3D | O95025 | 89.6 | 8.8 | 143.0 | 2.000 |
| gi\|105990532 | apolipoprotein B-100 precursor | APOB | P04114 | 515.2 | 6.6 | 135.2 | 2.000 |
| gi\|572882727 | 1-phosphatidylinositol 4,5-bisphosphate phosphodiesterase epsilon-1 isoform 3 | PLCE1 | Q9P212 | 256.9 | 6 | 106.8 | 2.000 |
| gi\|153791502 | transport and Golgi organization protein 6 homolog | TANGO6 | Q9C0B7 | 120.7 | 5.7 | 44.2 | 2.000 |
| gi\|544346335 | mitochondrial tRNA-specific 2-thiouridylase 1 isoform f | TRMU | O75648 | 26.9 | 10.3 | 35.9 | 2.000 |
| gi\|392050772 | zinc finger protein 850 isoform 2 | ZNF850 | A0A087X0M6 | 121.8 | 10.1 | 37.5 | 2.128 |
| gi\|519666794 | uromodulin isoform b preproprotein | UMOD | P07911 | 73.5 | 5 | 32.1 | 2.222 |
| gi\|4502157 | apolipoprotein C-I precursor | APOC1 | P02654 | 9.3 | 9.3 | 73.5 | 2.273 |
| gi\|545687541 | GMP reductase 2 isoform 3 | GMPR2 | Q9P2T1 | 40.9 | 9.4 | 40.5 | 2.273 |
| gi\|148886692 | protocadherin Fat 3 precursor | FAT3 | Q8TDW7 | 501.7 | 4.6 | 86.7 | 2.326 |
| gi\|7661998 | TBC1 domain family member 5 isoform b | TBC1D5 | Q92609 | 88.9 | 6.1 | 76.1 | 2.326 |
| gi\|154090976 | sterile alpha and TIR motif-containing protein 1 precursor | SARM1 | Q6SZW1 | 79.3 | 6.1 | 58.0 | 2.326 |
| gi\|555289992 | centrosomal protein of 162 kDa isoform b | CEP162 | Q5TB80 | 153 | 5.3 | 43.0 | 2.381 |
| gi\|157388933 | transmembrane protein 255A isoform 2 | TMEM255A | Q5JRV8 | 35.8 | 7.8 | 43.6 | 2.778 |
| gi\|284925165 | SUN domain-containing protein 1 isoform c | SUN1 | O94901 | 76.4 | 6.2 | 40.4 | 2.778 |
| gi\|14249340 | 1-phosphatidylinositol 4,5-bisphosphate phosphodiesterase delta-4 | PLCD4 | Q9BRC7 | 87.5 | 5 | 74.2 | 3.125 |
| gi\|7657303 | LIM/homeobox protein Lhx3 isoform b | LHX3 | Q9UBR4 | 43.9 | 9.5 | 82.6 | 5.263 |
| gi\|6912622 | DNA repair and recombination protein RAD54B isoform 1 | RAD54B | Q9Y620 | 102.9 | 9.4 | 98.7 | 8.333 |
