# Supplementary material for: Screening and Identification of APOC1 as a Novel Potential Biomarker for Differentiate of Mycoplasma pneumoniae in Children
Source: Front Microbiol. 2016 Dec 15;7:1961. doi: 10.3389/fmicb.2016.01961 (PMC5156883; doi:10.3389/fmicb.2016.01961)
Supplement: Supplementary file 4 [file Table4.DOCX]

**Supplementary 4: Classification of proteins identified following LC-MS/MS of MPP different from HC and IDC fractions.**

| **GO** | GO number | Category | No. | gene |
| --- | --- | --- | --- | --- |
| Biological Process | 0044085 | cellular component biogenesis | 8 | *ROCK1, KNTC1, GEMIN5, EXOC8, CFLAR, BRF1, APOC1, ANXA6* |
|  | 0032502 | developmental process | 5 | *ROCK1, YY1, CFLAR, RBBP6, PLCE1* |
|  | 0000003 | reproduction | 1 | *YY1* |
|  | 0016043 | cellular component organization | 14 | *SUN1, ROCK1, BAZ1A, ATXN7L3, CFLAR, MAP3K19, ANXA6, GEMIN5,YY1, BRF1, EXOC8, KNTC1, PLCE1, APOC1* |
|  | 0016265 | death | 2 | *CFLAR, ROCK1* |
|  | 0022414 | reproductive process | 1 | *YY1* |
|  | 0002376 | immune system process | 1 | *ROCK1* |
|  | 0050896 | response to stimulus | 5 | *YY1, RAD54B, ARPP21, CFLAR, SERPINA3* |
|  | 0032501 | multicellular organismal process | 8 | *APOC1, CFLAR, ROCK1, SERPINA3, PLCE1, ANXA6, RBBP6, YY1* |
|  | 0010926 | anatomical structure formation | 8 | *CFLAR, ROCK1, BRF1, EXOC8, APOC1, ANXA6, GEMIN5, KNTC1* |
|  | 0051234 | establishment of localization | 4 | *APOC1, ATP12A, EXOC8, ANXA6* |
|  | 0022610 | biological adhesion | 2 | *ROCK1, SDK2* |
|  | 0008152 | metabolic process | 18 | *PRDM15, ROCK1, CFLAR, ZNF850, BAZ1A, ATXN7L3, SERPINA3, MAP3K19, RBBP6, RAD54B, GEMIN5, YY1, ATP12A, USP17L10, BRF1, PLCE1, EXOC8, APOC1* |
|  | 0016032 | viral reproduction | 1 | *CFLAR* |
|  | 0043473 | pigmentation | 14 | *PRDM15, ROCK1, BAZ1A, ZNF850, CFLAR, ATXN7L3, SERPINA3, MAP3K19, ANXA6, YY1, BRF1, KNTC1, PLCE1, APOC1* |
|  | 0040011 | locomotion | 1 | *ROCK1* |
|  | 0051179 | localization | 6 | *ROCK1, EXOC8, YY1, APOC1, ATP12A, ANXA6* |
|  | 0009987 | cellular process | 22 | *ARPP21, SUN1, PRDM15, ROCK1, ZNF850, BAZ1A, ATXN7L3, CFLAR, RBBP6, MAP3K19, ANXA6, RAD54B, GEMIN5, ATP12A, USP17L10, YY1, BRF1, SDK2, KNTC1, EXOC8, PLCE1, APOC1* |
|  | 0040007 | growth | 3 | *PLCE1, CFLAR, RBBP6* |
|  | 0065007 | biological regulation | 15 | *PRDM15, ROCK1, BAZ1A, ZNF850, ATXN7L3, CFLAR, SERPINA3, MAP3K19, ANXA6, ATP12A, YY1, BRF1, PLCE1, KNTC1, APOC1* |
| Molecular Function | 0060089 | molecular transducer activity | 2 | *PLCE1, MAP3K19* |
|  | 0030528 | transcription regulator activity | 2 | *YY1, ATXN7L3* |
|  | 0030234 | enzyme regulator activity | 4 | *APOC1, SERPINA3, CFLAR, PLCE1* |
|  | 0003824 | catalytic activity | 9 | *USP17L10, ATP12A, PLCE1, RAD54B, CFLAR, PRDM15, ROCK1, MAP3K19, RBBP6* |
|  | 0005488 | binding | 23 | *ARPP21, SUN1, PRDM15, MTUS2, ROCK1, CFLAR, BAZ1A, ATXN7L3, ZNF850, SERPINA3, MAP3K19, RBBP6, RAD54B, ANXA6, GEMIN5, ATP12A, YY1, ZNF804B, BRF1, PLCE1, EXOC8, KNTC1, APOC1* |
|  | 0005215 | transporter activity | 2 | *ATP12A, ANXA6* |
| Cellular Component | 0005576 | extracellular region | 3 | SERPINA3, APOC1, ANXA6 |
|  | 0044421 | extracellular region part | 3 | SERPINA3, APOC1, ANXA6 |
|  | 0005623 | cell | 26 | ARPP21, SUN1, PRDM15, CCDC174, MTUS2, ROCK1, CFLAR, ZNF850, BAZ1A, ATXN7L3, SERPINA3, MAP3K19, RBBP6, RAD54B, ANXA6, GEMIN5, USP17L10, YY1, ATP12A, BRF1, CCDC88B, SDK2, PLCE1, EXOC8, KNTC1, APOC1 |
|  | 0044464 | cell part | 26 | ARPP21, SUN1, PRDM15, CCDC174, MTUS2, ROCK1, CFLAR, ZNF850, BAZ1A, ATXN7L3, SERPINA3, MAP3K19, RBBP6, RAD54B, ANXA6, GEMIN5, USP17L10, YY1, ATP12A, BRF1, CCDC88B, SDK2, PLCE1, EXOC8, KNTC1, APOC1 |
|  | 0031975 | envelope | 1 | SUN1 |
|  | 0032991 | macromolecular complex | 11 | APOC1, SUN1, GEMIN5, YY1, BAZ1A, CFLAR, EXOC8, BRF1, ATXN7L3, KNTC1, ATP12A |
|  | 0043226 | organelle | 19 | *SUN1, PRDM15, CCDC174, MTUS2, ROCK1, BAZ1A, ATXN7L3, SERPINA3, RBBP6, ANXA6, RAD54B, GEMIN5, YY1, USP17L10, BRF1, PLCE1, KNTC1, EXOC8, APOC1* |
|  | 0044422 | organelle part | 13 | *ANXA6, SUN1, ROCK1, PLCE1, ANXA6, CCDC174, RBBP6, GEMIN5, BRF1, ATXN7L3, YY1, RBBP6, KNTC1* |
|  | 0031974 | membrane-enclosed lumen | 6 | *CCDC174, RBBP6, GEMIN5, BRF1, ATXN7L3, YY1* |
