# Supplementary material for: Screening and Identification of APOC1 as a Novel Potential Biomarker for Differentiate of Mycoplasma pneumoniae in Children
Source: Front Microbiol. 2016 Dec 15;7:1961. doi: 10.3389/fmicb.2016.01961 (PMC5156883; doi:10.3389/fmicb.2016.01961)
Supplement: Supplementary file 5 [file Table5.DOCX]

**Supplementary 5: KEGG enrichment analysis of the identified proteins.**

| Term | Database | ID | Input number | Background number | *P*-Value | Input |
| --- | --- | --- | --- | --- | --- | --- |
| cAMP signaling pathway | KEGG PATHWAY | hsa04024 | 2 | 291 | 0.030601826 | Q13464\|Q9P212 |
| Proteoglycans in cancer | KEGG PATHWAY | hsa05205 | 2 | 294 | 0.031178826 | Q13464\|Q9P212 |
| Pathogenic Escherichia coli infection | KEGG PATHWAY | hsa05130 | 1 | 75 | 0.068671217 | Q13464 |
| Shigellosis | KEGG PATHWAY | hsa05131 | 1 | 91 | 0.082534162 | Q13464 |
| Inositol phosphate metabolism | KEGG PATHWAY | hsa00562 | 1 | 100 | 0.090243914 | Q9P212 |
| TGF-beta signaling pathway | KEGG PATHWAY | hsa04350 | 1 | 116 | 0.103795296 | Q13464 |
| Apoptosis | KEGG PATHWAY | hsa04210 | 1 | 122 | 0.10882643 | O15519 |
| Salmonella infection | KEGG PATHWAY | hsa05132 | 1 | 123 | 0.109662285 | Q13464 |
| NF-kappa B signaling pathway | KEGG PATHWAY | hsa04064 | 1 | 128 | 0.113830171 | O15519 |
| Phosphatidylinositol signaling system | KEGG PATHWAY | hsa04070 | 1 | 145 | 0.12785994 | Q9P212 |
| TNF signaling pathway | KEGG PATHWAY | hsa04668 | 1 | 149 | 0.131129626 | O15519 |
| Chagas disease | KEGG PATHWAY | hsa05142 | 1 | 159 | 0.139251921 | O15519 |
| Vascular smooth muscle contraction | KEGG PATHWAY | hsa04270 | 1 | 164 | 0.14328541 | Q13464 |
| Thyroid hormone signaling pathway | KEGG PATHWAY | hsa04919 | 1 | 164 | 0.14328541 | Q9P212 |
| Leukocyte transendothelial migration | KEGG PATHWAY | hsa04670 | 1 | 166 | 0.144893669 | Q13464 |
| Sphingolipid signaling pathway | KEGG PATHWAY | hsa04071 | 1 | 178 | 0.154481917 | Q13464 |
| Platelet activation | KEGG PATHWAY | hsa04611 | 1 | 180 | 0.156069789 | Q13464 |
| Axon guidance | KEGG PATHWAY | hsa04360 | 1 | 181 | 0.15686264 | Q13464 |
| Oxidative phosphorylation | KEGG PATHWAY | hsa00190 | 1 | 187 | 0.161604602 | P54707 |
| Oxytocin signaling pathway | KEGG PATHWAY | hsa04921 | 1 | 217 | 0.184929146 | Q13464 |
| MicroRNAs in cancer | KEGG PATHWAY | hsa05206 | 1 | 223 | 0.189517949 | Q13464 |
| RNA transport | KEGG PATHWAY | hsa03013 | 1 | 224 | 0.19028031 | Q8TEQ6 |
| cGMP-PKG signaling pathway | KEGG PATHWAY | hsa04022 | 1 | 240 | 0.202383929 | Q13464 |
| Calcium signaling pathway | KEGG PATHWAY | hsa04020 | 1 | 258 | 0.215790821 | Q9P212 |
| Chemokine signaling pathway | KEGG PATHWAY | hsa04062 | 1 | 263 | 0.219476008 | Q13464 |
| Rap1 signaling pathway | KEGG PATHWAY | hsa04015 | 1 | 295 | 0.242666641 | Q9P212 |
| Regulation of actin cytoskeleton | KEGG PATHWAY | hsa04810 | 1 | 305 | 0.249775677 | Q13464 |
| Focal adhesion | KEGG PATHWAY | hsa04510 | 1 | 310 | 0.253305887 | Q13464 |
| Ras signaling pathway | KEGG PATHWAY | hsa04014 | 1 | 317 | 0.25822113 | Q9P212 |
| Pathways in cancer | KEGG PATHWAY | hsa05200 | 1 | 578 | 0.420681797 | Q13464 |
| Metabolic pathways | KEGG PATHWAY | hsa01100 | 1 | 1709 | 0.805831521 | Q9P212 |
