# Supplementary material for: Screening and Identification of APOC1 as a Novel Potential Biomarker for Differentiate of Mycoplasma pneumoniae in Children
Source: Front Microbiol. 2016 Dec 15;7:1961. doi: 10.3389/fmicb.2016.01961 (PMC5156883; doi:10.3389/fmicb.2016.01961)
Supplement: Supplementary file 6 [file Table6.DOCX]

**Supplementary 6: ELISA results of plasma protein levels in the MPP (n = 85), HC (n = 95), and IDC (n = 75) groups.**

|  | SERPINA3 | ANXA6 | APOC1 | CFLAR | KNTC1 |  | SERPINA3 | ANXA6 | APOC1 | CFLAR | KNTC1 |  | SERPINA3 | ANXA6 | APOC1 | CFLAR | KNTC1 |
| --- | --- | --- | --- | --- | --- | --- | --- | --- | --- | --- | --- | --- | --- | --- | --- | --- | --- |
| MPP1 | 252.88 | 0.09 | 309.40 | 0.43 | 0.66 | HC1 | 343.02 | 5.05 | 176.10 | 0.44 | 1.38 | IDC1 | 378.42 | 2.19 | 231.92 | 0.67 | 1.52 |
| MPP2 | 323.92 | 0.09 | 414.07 | 0.57 | 0.97 | HC2 | 398.71 | 7.97 | 166.98 | 0.95 | 0.73 | IDC2 | 443.10 | 7.81 | 188.62 | 0.86 | 1.01 |
| MPP3 | 324.72 | 0.36 | 455.97 | 1.58 | 0.27 | HC3 | 436.60 | 4.14 | 200.67 | 0.79 | 0.86 | IDC3 | 621.40 | 0.52 | 217.99 | 1.64 | 0.74 |
| MPP4 | 297.05 | 0.18 | 376.98 | 0.47 | 0.52 | HC4 | 653.00 | 5.45 | 157.46 | 4.37 | 1.50 | IDC4 | 433.42 | 0.52 | 222.92 | 1.15 | 0.00 |
| MPP5 | 259.12 | 0.07 | 355.75 | 0.25 | 0.18 | HC5 | 484.06 | 4.63 | 191.81 | 0.83 | 1.26 | IDC5 | 217.38 | 0.69 | 219.92 | 0.75 | 0.48 |
| MPP6 | 195.56 | 0.09 | 245.93 | 1.16 | 0.26 | HC6 | 413.31 | 4.17 | 240.94 | 0.43 | 1.62 | IDC6 | 406.63 | 0.49 | 213.70 | 0.40 | 0.33 |
| MPP7 | 233.28 | 0.10 | 383.27 | 0.87 | 1.31 | HC7 | 533.79 | 3.55 | 207.66 | 0.46 | 1.28 | IDC7 | 289.92 | 0.13 | 124.01 | 0.61 | 1.11 |
| MPP8 | 232.95 | 0.07 | 305.96 | 0.79 | 0.29 | HC8 | 443.97 | 1.98 | 231.13 | 1.18 | 1.82 | IDC8 | 392.57 | 0.77 | 136.52 | 0.58 | 1.54 |
| MPP9 | 233.16 | 0.10 | 381.32 | 0.29 | 1.00 | HC9 | 149.83 | 3.83 | 41.47 | 0.88 | 1.43 | IDC9 | 187.38 | 0.22 | 187.28 | 1.05 | 0.36 |
| MPP10 | 296.25 | 6.07 | 416.02 | 0.59 | 0.44 | HC10 | 462.88 | 5.04 | 245.01 | 2.79 | 0.14 | IDC10 | 654.27 | 1.32 | 185.53 | 0.49 | 0.00 |
| MPP11 | 216.85 | 0.33 | 334.07 | 0.56 | 1.02 | HC11 | 541.35 | 4.16 | 179.00 | 0.39 | 1.53 | IDC11 | 298.16 | 1.32 | 182.12 | 0.33 | 1.64 |
| MPP12 | 192.20 | 0.03 | 245.30 | 0.04 | 0.61 | HC12 | 521.79 | 2.50 | 201.08 | 0.37 | 1.14 | IDC12 | 357.84 | 0.52 | 173.11 | 1.37 | 1.53 |
| MPP13 | 293.25 | 0.08 | 292.00 | 0.35 | 1.85 | HC13 | 473.22 | 1.86 | 240.01 | 0.30 | 1.31 | IDC13 | 348.27 | 0.51 | 167.31 | 2.65 | 1.09 |
| MPP14 | 273.72 | 0.11 | 338.44 | 0.56 | 0.22 | HC14 | 454.81 | 5.44 | 159.94 | 3.91 | 0.00 | IDC14 | 332.68 | 0.82 | 197.82 | 0.31 | 1.18 |
| MPP15 | 307.46 | 0.07 | 216.23 | 0.28 | 1.16 | HC15 | 382.24 | 4.17 | 67.18 | 0.74 | 1.10 | IDC15 | 401.68 | 0.67 | 232.28 | 1.01 | 0.43 |
| MPP16 | 166.37 | 0.27 | 345.63 | 0.42 | 0.46 | HC16 | 398.16 | 3.23 | 225.72 | 0.96 | 1.46 | IDC16 | 399.27 | 0.54 | 190.95 | 0.47 | 0.00 |
| MPP17 | 262.05 | 0.15 | 323.28 | 0.96 | 0.26 | HC17 | 192.28 | 2.72 | 224.14 | 0.68 | 1.61 | IDC17 | 385.43 | 0.24 | 168.74 | 0.74 | 0.00 |
| MPP18 | 212.09 | 0.07 | 351.13 | 0.21 | 0.00 | HC18 | 380.31 | 5.46 | 219.52 | 0.32 | 1.96 | IDC18 | 316.90 | 0.53 | 157.73 | 0.68 | 2.45 |
| MPP19 | 234.36 | 0.21 | 284.54 | 0.20 | 1.18 | HC19 | 504.49 | 4.86 | 239.47 | 0.44 | 1.36 | IDC19 | 530.18 | 0.37 | 191.19 | 0.59 | 0.00 |
| MPP20 | 276.27 | 0.41 | 312.77 | 1.71 | 1.15 | HC20 | 439.75 | 1.68 | 185.96 | 0.34 | 0.73 | IDC20 | 519.07 | 4.10 | 200.66 | 0.90 | 1.09 |
| MPP21 | 243.16 | 13.24 | 412.70 | 1.81 | 0.73 | HC21 | 330.94 | 0.93 | 119.11 | 0.34 | 2.16 | IDC21 | 485.67 | 1.26 | 117.90 | 2.79 | 0.41 |
| MPP22 | 308.03 | 0.08 | 114.55 | 0.74 | 0.66 | HC22 | 389.24 | 4.55 | 271.84 | 2.73 | 1.18 | IDC22 | 221.61 | 0.66 | 201.69 | 0.56 | 0.29 |
| MPP23 | 139.70 | 0.32 | 123.72 | 0.14 | 1.02 | HC23 | 328.15 | 3.91 | 173.82 | 0.49 | 1.37 | IDC23 | 373.25 | 0.00 | 175.58 | 0.04 | 0.45 |
| MPP24 | 137.51 | 0.01 | 175.75 | 0.33 | 0.65 | HC24 | 333.29 | 4.39 | 216.87 | 0.83 | 1.56 | IDC24 | 309.26 | 0.84 | 85.17 | 1.72 | 0.93 |
| MPP25 | 184.12 | 0.02 | 187.92 | 1.11 | 0.54 | HC25 | 217.61 | 2.71 | 154.87 | 0.67 | 1.19 | IDC25 | 431.30 | 0.60 | 186.93 | 0.31 | 0.29 |
| MPP26 | 231.89 | 0.09 | 185.80 | 0.48 | 0.79 | HC26 | 409.18 | 3.36 | 251.50 | 0.59 | 2.12 | IDC26 | 512.55 | 0.17 | 166.88 | 2.18 | 0.79 |
| MPP27 | 260.34 | 0.04 | 260.93 | 0.77 | 0.78 | HC27 | 439.31 | 5.92 | 250.18 | 0.43 | 1.73 | IDC27 | 396.54 | 0.49 | 107.70 | 1.58 | 0.00 |
| MPP28 | 220.64 | 0.03 | 269.90 | 0.71 | 0.81 | HC28 | 383.65 | 2.82 | 146.77 | 1.81 | 1.27 | IDC28 | 267.98 | 0.08 | 143.96 | 3.01 | 0.02 |
| MPP29 | 216.06 | 0.06 | 214.00 | 0.56 | 0.49 | HC29 | 326.18 | 2.96 | 147.66 | 0.82 | 1.11 | IDC29 | 318.46 | 0.53 | 80.84 | 1.08 | 2.60 |
| MPP30 | 176.77 | 0.01 | 229.31 | 0.56 | 0.00 | HC30 | 405.13 | 2.07 | 264.22 | 2.53 | 1.38 | IDC30 | 448.11 | 1.00 | 144.97 | 2.19 | 0.00 |
| MPP31 | 146.04 | 0.02 | 296.61 | 0.41 | 0.61 | HC31 | 425.40 | 2.89 | 189.04 | 0.70 | 1.79 | IDC31 | 406.95 | 0.22 | 133.07 | 2.97 | 0.56 |
| MPP32 | 120.92 | 0.05 | 167.50 | 1.34 | 0.82 | HC32 | 286.12 | 3.19 | 169.55 | 0.35 | 0.00 | IDC32 | 470.64 | 2.49 | 240.09 | 1.05 | 0.66 |
| MPP33 | 211.80 | 0.13 | 239.03 | 0.83 | 0.00 | HC33 | 243.70 | 8.27 | 107.95 | 0.11 | 0.86 | IDC33 | 390.73 | 6.22 | 189.38 | 0.22 | 0.98 |
| MPP34 | 229.99 | 0.07 | 242.75 | 0.24 | 0.82 | HC34 | 376.18 | 6.46 | 179.24 | 0.00 | 1.77 | IDC34 | 557.81 | 0.32 | 221.44 | 19.69 | 0.00 |
| MPP35 | 141.74 | 0.05 | 213.27 | 0.56 | 0.67 | HC35 | 382.59 | 9.11 | 166.34 | 0.16 | 0.00 | IDC35 | 87.49 | 0.97 | 83.94 | 1.50 | 1.90 |
| MPP36 | 151.24 | 0.10 | 243.37 | 0.39 | 0.31 | HC36 | 409.67 | 2.42 | 165.52 | 0.01 | 0.70 | IDC36 | 373.11 | 8.54 | 78.88 | 0.54 | 0.38 |
| MPP37 | 121.90 | 0.12 | 292.16 | 0.83 |  | HC37 | 483.13 | 1.48 | 187.70 | 0.11 | 0.00 | IDC37 | 424.92 | 0.54 | 124.15 | 2.04 | 4.04 |
| MPP38 | 172.07 | 0.11 | 222.83 | 1.01 | 0.72 | HC38 | 401.06 | 2.93 | 185.98 | 0.13 | 1.69 | IDC38 | 360.88 | 0.30 | 126.16 | 3.17 | 0.46 |
| MPP39 | 283.72 | 0.02 | 342.43 | 0.35 | 0.16 | HC39 | 283.90 | 11.00 | 68.31 | 1.69 | 0.00 | IDC39 | 505.86 | 0.47 | 206.69 | 1.58 | 0.00 |
| MPP40 | 203.80 | 0.07 | 307.01 | 0.30 | 0.00 | HC40 | 214.17 | 2.71 | 140.75 | 3.50 | 0.71 | IDC40 | 180.90 | 0.42 | 132.52 | 0.61 | 0.00 |
| MPP41 | 243.51 | 0.12 | 400.74 | 0.33 | 1.20 | HC41 | 392.89 | 3.14 | 181.87 | 0.22 | 1.89 | IDC41 | 391.16 | 0.43 | 184.73 | 0.56 | 1.41 |
| MPP42 | 218.27 | 0.12 | 407.03 | 0.15 | 0.40 | HC42 | 330.39 | 3.14 | 163.44 | 1.52 | 0.86 | IDC42 | 300.46 | 0.91 | 198.79 | 4.18 | 0.93 |
| MPP43 | 218.22 | 0.14 | 340.27 | 0.50 | 1.11 | HC43 | 414.30 | 5.64 | 173.69 | 0.30 | 0.46 | IDC43 | 410.76 | 0.67 | 190.18 | 0.27 | 0.66 |
| MPP44 | 194.14 | 0.17 | 437.98 | 0.59 | 0.69 | HC44 | 484.80 | 3.22 | 167.43 | 1.87 | 0.00 | IDC44 | 431.81 | 0.47 | 93.10 | 0.49 | 1.52 |
| MPP45 | 287.34 | 0.19 | 336.20 | 0.43 | 3.76 | HC45 | 438.64 | 1.32 | 206.02 | 0.14 | 0.07 | IDC45 | 399.79 | 0.87 | 156.65 | 7.52 | 0.72 |
| MPP46 | 211.18 | 0.06 | 290.57 | 0.51 | 0.38 | HC46 | 308.95 | 5.24 | 166.16 | 1.66 | 1.19 | IDC46 | 385.40 | 0.19 | 79.22 | 0.47 | 0.72 |
| MPP47 | 241.18 | 0.02 | 297.03 | 0.07 | 0.03 | HC47 | 341.58 | 5.64 | 202.23 | 1.02 | 1.18 | IDC47 | 294.18 | 0.24 | 84.26 | 2.65 | 1.56 |
| MPP48 | 204.11 | 4.77 | 243.01 | 0.79 | 0.97 | HC48 | 418.42 | 1.66 | 156.10 | 0.31 | 0.89 | IDC48 | 549.49 | 9.47 | 88.39 | 1.49 | 1.84 |
| MPP49 | 351.06 | 0.62 | 197.33 | 0.74 | 0.48 | HC49 | 270.87 | 2.62 | 158.59 | 0.46 | 0.00 | IDC49 | 167.55 | 0.09 | 344.76 | 0.29 | 0.73 |
| MPP50 | 578.36 | 43.69 | 218.92 | 0.52 | 3.09 | HC50 | 385.95 | 3.34 | 152.73 | 1.13 | 0.59 | IDC50 | 139.54 | 0.02 | 170.60 | 1.34 | 0.35 |
| MPP51 | 413.30 | 0.65 | 160.76 | 0.42 | 0.37 | HC51 | 371.37 | 4.00 | 173.85 | 0.28 | 0.46 | IDC51 | 171.94 | 0.04 | 212.35 | 0.12 | 0.00 |
| MPP52 | 297.44 | 1.04 | 246.07 | 0.55 | 0.31 | HC52 | 505.24 | 5.77 | 162.17 | 0.41 | 0.02 | IDC52 | 359.47 | 0.34 | 221.53 | 1.23 | 0.89 |
| MPP53 | 169.83 | 2.05 | 245.77 | 1.31 | 0.55 | HC53 | 483.95 | 1.93 | 118.03 | 1.28 | 0.00 | IDC53 | 385.44 | 0.90 | 186.40 | 0.26 | 0.61 |
| MPP54 | 375.14 | 2.27 | 194.13 | 0.62 | 1.70 | HC54 | 379.97 | 2.64 | 191.14 | 0.14 | 0.00 | IDC54 | 292.22 | 0.35 | 169.93 | 0.41 | 0.29 |
| MPP55 | 394.72 | 1.65 | 262.99 | 0.65 | 1.01 | HC55 | 388.98 | 3.71 | 219.17 | 2.01 | 0.70 | IDC55 | 461.19 | 0.50 | 204.41 | 0.03 | 0.69 |
| MPP56 | 488.42 | 0.28 | 254.46 | 0.61 | 0.00 | HC56 | 193.19 | 1.99 | 108.24 | 0.00 | 1.43 | IDC56 | 486.78 | 1.31 | 185.62 | 0.20 | 0.70 |
| MPP57 | 246.84 | 0.16 | 240.94 | 0.38 | 2.32 | HC57 | 333.54 | 1.27 | 213.25 | 0.22 | 1.16 | IDC57 | 201.38 | 0.49 | 73.15 | 4.16 | 0.65 |
| MPP58 | 251.32 | 4.22 | 226.52 | 0.84 | 0.37 | HC58 | 264.86 | 2.22 | 175.10 | 0.38 | 0.59 | IDC58 | 234.39 | 0.40 | 79.06 | 0.62 | 1.56 |
| MPP59 | 406.48 | 1.89 | 187.81 | 0.49 | 0.00 | HC59 | 381.65 | 2.76 | 185.33 | 0.15 | 0.73 | IDC59 | 141.93 | 0.23 | 128.11 | 0.70 | 0.18 |
| MPP60 | 475.48 | 0.45 | 216.32 | 0.53 | 0.00 | HC60 | 466.11 | 5.27 | 165.13 | 10.88 | 1.89 | IDC60 | 321.77 | 0.15 | 90.20 | 0.28 | 0.00 |
| MPP61 | 556.98 | 0.92 | 233.64 | 0.35 | 0.00 | HC61 | 470.26 | 3.48 | 214.43 | 4.83 | 0.04 | IDC61 | 377.91 | 0.24 | 177.41 | 0.27 | 0.79 |
| MPP62 | 382.95 | 1.50 | 214.52 | 2.06 | 0.00 | HC62 | 429.94 | 5.15 | 147.44 | 1.85 | 0.30 | IDC62 | 200.40 | 0.53 | 67.90 | 1.46 | 1.91 |
| MPP63 | 321.83 | 0.70 | 217.55 | 1.12 | 0.39 | HC63 | 328.72 | 4.57 | 170.97 | 2.35 | 0.00 | IDC63 | 165.82 | 0.37 | 97.85 | 0.87 | 0.57 |
| MPP64 | 282.28 | 2.68 | 258.21 | 0.59 | 0.04 | HC64 | 362.07 | 13.64 | 23.64 | 0.08 | 0.00 | IDC64 | 159.16 | 0.16 | 77.44 | 1.13 | 1.18 |
| MPP65 | 280.80 | 1.59 | 221.90 | 0.42 | 0.75 | HC65 | 230.17 | 1.02 | 148.09 | 0.26 | 0.46 | IDC65 | 353.89 | 1.17 | 104.24 | 0.33 | 1.16 |
| MPP66 | 343.94 | 2.08 | 271.24 | 0.53 | 2.25 | HC66 | 252.90 | 1.61 | 124.52 | 0.26 | 0.88 | IDC66 | 340.10 | 1.22 | 75.70 | 4.90 | 1.43 |
| MPP67 | 189.44 | 0.65 | 247.29 | 0.46 | 0.50 | HC67 | 300.89 | 1.44 | 202.27 | 0.47 | 0.93 | IDC67 | 379.84 | 0.70 | 203.40 | 0.63 | 1.36 |
| MPP68 | 186.34 | 0.59 | 113.77 | 1.37 | 0.53 | HC68 | 402.67 | 1.45 | 193.09 | 0.90 | 1.46 | IDC68 | 164.59 | 0.33 | 67.15 | 2.48 | 1.62 |
| MPP69 | 462.46 | 1.39 | 232.38 | 0.59 | 0.33 | HC69 | 364.35 | 0.00 | 176.64 | 0.53 | 2.39 | IDC69 | 210.63 | 0.16 | 125.75 | 3.05 | 1.89 |
| MPP70 | 567.69 | 1.77 | 298.22 | 0.72 | 2.07 | HC70 | 292.26 | 0.00 | 234.58 | 0.53 | 0.86 | IDC70 | 360.50 | 0.74 | 202.70 | 1.44 | 0.00 |
| MPP71 | 411.29 | 0.70 | 195.20 | 0.56 | 0.49 | HC71 | 298.28 | 0.36 | 220.09 | 0.00 | 0.84 | IDC71 | 391.64 | 0.11 | 90.60 | 0.28 | 2.12 |
| MPP72 | 250.17 | 1.55 | 195.47 | 0.74 | 0.00 | HC72 | 317.32 | 0.18 | 200.11 | 0.48 | 1.43 | IDC72 | 149.60 | 0.04 | 80.78 | 0.70 | 0.00 |
| MPP73 | 251.54 | 0.23 | 175.68 | 1.22 | 0.42 | HC73 | 226.43 | 0.81 | 164.38 | 0.24 | 1.09 | IDC73 | 50.14 | 0.05 | 79.96 | 2.88 | 0.58 |
| MPP74 | 187.59 | 1.25 | 174.43 | 0.40 | 4.12 | HC74 | 265.67 | 0.77 | 166.20 | 0.24 | 1.00 | IDC74 | 94.89 | 0.05 | 79.15 | 0.48 | 1.37 |
| MPP75 | 147.38 | 0.35 | 234.86 | 0.45 | 1.00 | HC75 | 261.20 | 2.04 | 147.82 | 0.99 | 0.46 | IDC75 | 151.73 | 0.14 | 113.40 | 1.00 | 1.31 |
| MPP76 | 299.21 | 1.04 | 226.61 | 0.45 | 2.14 | HC76 | 276.06 | 1.65 | 167.62 | 0.99 | 0.75 |  |  |  |  |  |  |
| MPP77 | 543.90 | 0.58 | 255.00 | 0.76 | 0.53 | HC77 | 364.58 | 0.00 | 184.69 | 1.55 | 1.55 |  |  |  |  |  |  |
| MPP78 | 318.70 | 0.62 | 157.00 | 0.40 | 0.00 | HC78 | 307.28 | 1.00 | 174.78 | 0.21 | 0.00 |  |  |  |  |  |  |
| MPP79 | 512.74 | 0.54 | 263.29 | 0.81 | 0.86 | HC79 | 276.77 | 1.37 | 180.74 | 1.24 | 1.10 |  |  |  |  |  |  |
| MPP80 | 481.85 | 8.81 | 212.58 | 1.18 | 0.86 | HC80 | 202.79 | 0.67 | 211.83 | 1.08 | 1.09 |  |  |  |  |  |  |
| MPP81 | 204.81 | 1.88 | 170.45 | 0.40 | 0.19 | HC81 | 297.27 | 2.61 | 152.58 | 5.27 | 1.59 |  |  |  |  |  |  |
| MPP82 | 274.85 | 1.05 | 250.82 | 0.47 | 1.17 | HC82 | 344.62 | 0.22 | 144.77 | 0.48 | 1.19 |  |  |  |  |  |  |
| MPP83 | 457.57 | 1.86 | 258.40 | 1.48 | 0.55 | HC83 | 363.44 | 1.52 | 181.54 | 3.48 | 0.00 |  |  |  |  |  |  |
| MPP84 | 463.94 | 1.30 | 158.65 | 0.74 | 0.09 | HC84 | 284.19 | 0.50 | 153.19 | 2.26 | 1.66 |  |  |  |  |  |  |
| MPP85 | 230.22 | 0.54 | 192.18 | 0.77 | 0.42 | HC85 | 277.89 | 0.80 | 187.63 | 0.23 | 2.67 |  |  |  |  |  |  |
|  |  |  |  |  |  | HC86 | 365.33 | 0.00 | 177.61 | 0.89 | 0.66 |  |  |  |  |  |  |
|  |  |  |  |  |  | HC87 | 294.61 | 0.80 | 172.14 | 0.41 | 1.07 |  |  |  |  |  |  |
|  |  |  |  |  |  | HC88 | 202.37 | 1.72 | 171.97 | 0.17 | 0.75 |  |  |  |  |  |  |
|  |  |  |  |  |  | HC89 | 200.26 | 1.34 | 95.47 | 0.43 | 1.21 |  |  |  |  |  |  |
|  |  |  |  |  |  | HC90 | 388.21 | 1.20 | 119.15 | 1.76 | 0.86 |  |  |  |  |  |  |
|  |  |  |  |  |  | HC91 | 391.91 | 3.23 | 94.13 | 3.37 | 0.79 |  |  |  |  |  |  |
|  |  |  |  |  |  | HC92 | 380.17 | 0.70 | 135.83 | 1.16 | 1.12 |  |  |  |  |  |  |
|  |  |  |  |  |  | HC93 | 489.64 | 0.55 | 109.84 | 1.16 | 1.65 |  |  |  |  |  |  |
|  |  |  |  |  |  | HC94 | 512.10 | 0.00 | 100.29 | 0.63 | 1.10 |  |  |  |  |  |  |
|  |  |  |  |  |  | HC95 | 388.68 | 0.57 | 155.98 | 0.59 | 1.44 |  |  |  |  |  |  |
